# Citrate-Mediated Acyl-CoA Synthesis Is Required for the Promotion of Growth and Triacylglycerol Production in Oleaginous Yeast *Lipomyces starkeyi*

**DOI:** 10.3390/microorganisms9081693

**Published:** 2021-08-09

**Authors:** Rikako Sato, Satoshi Ara, Harutake Yamazaki, Koji Ishiya, Sachiyo Aburatani, Hiroaki Takaku

**Affiliations:** 1 Department of Applied Life Sciences, Niigata University of Pharmacy and Applied Life Sciences, Niigata 956-8603, Japan; sato.rikako03@gmail.com (R.S.); ara@nupals.ac.jp (S.A.); hyamazaki@nupals.ac.jp (H.Y.); 2 Bioproduction Research Institute (BPRI), National Institute of Advance Industrial Science and Technology (AIST), Sapporo 062-8517, Japan; koji.ishiya@aist.go.jp; 3 Computational Bio Big-Data Open Innovation Laboratory (CBBD-OIL), National Institute of Advanced Industrial Science and Technology (AIST), Tokyo 135-0064, Japan

**Keywords:** oleaginous yeast, triacylglycerol, acyl-CoA synthesis, ATP-citrate lyase, *Lipomyces starkeyi*

## Abstract

The oleaginous yeast *Lipomyces starkeyi* is an excellent producer of triacylglycerol (TAG) as a feedstock for biodiesel production. To understand the regulation of TAG synthesis, we attempted to isolate mutants with decreased lipid productivity and analyze the expression of TAG synthesis-related genes in this study. A mutant with greatly decreased lipid productivity, sr22, was obtained by an effective screening method using Percoll density gradient centrifugation. The expression of citrate-mediated acyl-CoA synthesis-related genes (*ACL1*, *ACL2*, *ACC1*, *FAS1*, and *FAS2*) was decreased in the sr22 mutant compared with that of the wild-type strain. Together with a notion that *L. starkeyi* mutants with increased lipid productivities had increased gene expression, there was a correlation between the expression of these genes and TAG synthesis. To clarify the importance of citrate-mediated acyl-CoA synthesis pathway on TAG synthesis, we also constructed a strain with no ATP-citrate lyase responsible for the first reaction of citrate-mediated acyl-CoA synthesis and investigated the importance of ATP-citrate lyase on TAG synthesis. The ATP-citrate lyase was required for the promotion of cell growth and TAG synthesis in a glucose medium. This study may provide opportunities for the development of an efficient TAG synthesis for biodiesel production.

## 1. Introduction

The microbial lipids produced by oleaginous microorganisms are called single cell oils (SCO), which are potential alternatives for the production of edible oils, oleochemicals, and biofuels such as biodiesel. Oleaginous microorganisms, including bacteria, yeast, molds, and microalgae, can accumulate lipids up to 20% of their dry cell weight [1]. In recent years, the production of cocoa butter-like lipid by SCO replaces the expensive materials, such as cocoa butter, and their accumulated lipids are expected to play an important role as functional lipids for human health, such as γ-linoleic acid (GLA), arachidonic acid (ARA), docosahexaenoic acid (DHA), and eicosapentaenoic acid (EPA) [2]. Oleaginous yeasts (e.g., *Cryptococcus curvatus*, *Lipomyces starkeyi*, *Rhodosporidium toruloides*, *Rhodotorula glutinis*, *R. graminis*, and *Yarrowia lipolytica*) can accumulate lipids mainly as triglycerides (TAGs) up to 30–70% of their dry cell weight, which are similar to the fatty acid profiles of vegetable oils [1]. Among these oleaginous yeasts, *L. starkeyi* is an attractive oil producer with high lipid accumulation for industrial use potential. *L. starkeyi* NBRC10381 accumulated lipids up to 85% of their dry cell weight, with high inoculum sizes under nitrogen-limited conditions [3]. *L. starkeyi* can convert various carbon sources, such as glucose, xylose, arabinose, galactose, mannose, and cellobiose, into energy and lipid production [4]. In addition, *L. starkeyi* can assimilate waste and raw materials (e.g., crude glycerol) [5,6] and agricultural residues (e.g., rice straw) [7], which leads to avoidance of competition with edible substrates.

Oleaginous yeasts are known to accumulate lipids when there is excess carbon in a nitrogen-limited or other nutrient-limited culture [1,8]. Therefore, the ratio of carbon sources and nitrogen sources (C/N ratio) is an important factor for lipid accumulation [9]. The lipid accumulation in *L. starkeyi* is affected by the C/N ratio, aeration levels, pH, and alcohol byproducts [10]. Recently, the genome of *L. starkeyi* NRRL Y-11557 was published [11], and its genome sequence will facilitate the engineering of lipid biosynthetic and degradative pathways with genomic tools. Furthermore, optimized genetic transformation systems [12,13,14,15], development of a host designed for highly efficient gene targeting [16], and overexpression via multiple integration of the target gene into an rDNA locus [13] were established for *L. starkeyi*. The optimized lipid production by metabolic engineering, with available information and genetic engineering tools, will potentially lead to an economically competitive production of biodiesel and oleochemicals using *L. starkeyi*.

Random mutagenesis treatment with mutagens, such as ethyl methanesulfonate, nitrosoguanidine (NTG), and ultraviolet (UV) irradiation, improved the lipid productivity of several oleaginous yeasts, such as *R. glutinis* [17], *R. toruloides* [18,19,20], *Y. lipolytica* [21,22], *L. starkeyi* [23,24,25], and *C. curvatus* [26]. The transcriptional analysis of the isolated mutants with high levels of lipid production revealed the genes influencing the lipid production [19,20,25,26]. The isolation and characterization of mutants with altered lipid productivity provide us with valuable factors to improve it. As mentioned above, there are many studies on the acquisition and analysis of mutants with high lipid productivity, but a mutant with decreased lipid productivity has never been isolated in oleaginous yeasts. Although the positive information obtained from high lipid productivity strains is useful to clarify the mechanism of TAG synthesis in *L. starkeyi*, the identification of genes responsible for the decreased lipid productivity and obtaining the negative information are important.

To understand the regulation of TAG synthesis in *L. starkeyi*, the mutants with significantly decreased TAG productivity were isolated and characterized in this study. Wild-type *L. starkeyi* CBS1807 cells were mutagenized using UV irradiation, followed by Percoll density gradient centrifugation for the enrichment of high-density cells that were expected to accumulate small amounts of TAG. The sr22 mutant with defective TAG synthesis was isolated from the high-density fraction. Then, the gene expression of citrate-mediated acyl-CoA synthesis pathway on TAG synthesis was evaluated in the wild-type strain and sr22 mutant. Furthermore, we constructed *L. starkeyi* strains carrying deleted or highly expressed *ACL1* and *ACL2* genes coding for ATP-citrate lyase (ACL), which is responsible for the first reaction of citrate-mediated acyl-CoA synthesis pathway, and we revealed the importance of citrate-mediated acyl-CoA synthesis in the growth and TAG synthesis of *L. starkeyi*.

## 2. Materials and Methods

### 2.1. Strain and Media

The bacterial and yeast strains used in this study are listed in Table S1. *Lipomyces starkeyi* CBS1807 (Centraalbureau voor Schimmelcultures) was chosen as the wild-type strain for mutagenesis based on our previous study [24]. The ∆*lslig4* strain is a nonhomologous end-joining-deficient strain with highly efficient homologous recombination. The ∆*lslig4* was generated from *L. starkeyi* strain CBS1807 [16]. The yeast cells were cultured in a yeast extract/peptone/dextrose (YPD) medium (1% yeast extract (Kyokutou, Tokyo, Japan), 2% HIPOLYPEPTON (Nihon Pharmaceutical, Tokyo, Japan), and 2% glucose), semidefined medium (S medium) (0.5% (NH_4_)_2_SO_4_, 0.1% KH_2_PO_4_, 0.01% NaCl, 0.1% yeast extract (Kyokutou, Tokyo, Japan), 0.05% MgSO_4_·7H_2_O, 0.01% CaCl_2_·2H_2_O, and 5% glucose), and semidefined glycerol medium (SG medium) (0.5% (NH_4_)_2_SO_4_, 0.1% KH_2_PO_4_, 0.01% NaCl, 0.1% yeast extract (Kyokutou, Tokyo, Japan), 0.05% MgSO_4_·7H_2_O, 0.01% CaCl_2_·2H_2_O, and 2% glycerol) at 30 °C, as previously described [24]. The S and SG media were adjusted to pH 5.5 by adding 1 N NaOH. To the solid medium, 2% agar (FUJIFILM Wako Pure Chemical, Osaka, Japan) was added. *Escherichia coli* HST08 cells were grown at 37 °C in a liquid L-broth medium with 100 µg/mL ampicillin medium (1% Bacto^TM^ Tryptone (BD Biosciences, Franklin Lakes, NJ, USA), 0.5% Bacto^TM^ Yeast Extract (BD Biosciences), and 0.5% NaCl).

### 2.2. Determination of Cell Concentration

The cell cultures were adequately diluted with Cell Pack (Sysmex, Kobe, Japan) and sonicated with a Vibra Cell sonicator (Sonics & Materials, Danbury, CT, USA) for 20 s (cycles of 5 s on and 5 s off). The number and diameter of *L. starkeyi* cells cultures were measured using a CDA-1000 electronic particle counter (Sysmex).

### 2.3. UV Treatment

The UV treatment of *L. starkeyi* CBS1807 cells was performed only once before the first Percoll gradient centrifugation, as previously described [25], with an UV irradiation time of 35 s. The cells were obtained, washed with SG medium, and resuspended in 1 mL of SG medium. High-density cell enrichment was performed using Percoll density gradient centrifugation.

### 2.4. Cell Survival Rate after UV Treatment

The UV-treated (0.816 mW/cm^2^) and untreated cell suspensions were diluted to 2000 and 10,000 cells/mL with a YPD medium, and 100 µL of each diluted cell suspension were inoculated on a YPD solid medium. After four days of incubation at 30 °C, the number of colonies was counted, and the survival rate of the UV-treated cells was calculated as follows: survival rate (%) = (colony count of UV-treated cells)/(colony count of untreated cells) × 100.

### 2.5. Enrichment of High-Density Cells Using Percoll Density Gradient Centrifugation

The UV-treated cells were enriched using previously described techniques [24]. Briefly, the UV-treated cells, other than those used for the measurement of cell survival rate, were washed once with an SG medium; then, the cells were inoculated with 50 mL of SG medium in a 200 mL baffled flask and cultured for six days at 30 °C and 160 rpm. The cultured cells were inoculated at 3.0 × 10^6^ cells/mL into 300 mL of S medium in a 500 mL baffled flask and cultured for one day at 30 °C and 160 rpm to prepare cells in log phase with glucose assimilation and little accumulation of TAG. Then, the resultant culture was again inoculated at 1.2 × 10^7^ cells/mL into 300 mL of S medium in a 500 mL baffled flask and cultured for six days at 30 °C and 160 rpm. About 1.0 × 10^9^ cells were obtained from the culture via centrifugation at 2270× *g* for 5 min at 25 °C. After removing the supernatant, the cells were suspended with 1 mL of PBS (0.8% NaCl, 0.29% Na_2_HPO_4_·12H_2_O, 0.02% KCl, and 0.02% KH_2_PO_4_). This cell suspension was mixed with 8 mL of 90% Percoll (Cytiva, Uppsala, Sweden) and centrifuged at 22,000 rpm in a 70.1 Ti rotor (Beckman Coulter, Brea, CA, USA) for 20 min at 25 °C. After the Percoll density gradient centrifugation, approximately 3 mL of the lower fraction containing the high-density cells was fractionated and inoculated on an SG medium for the next cultivation. This enrichment of high-density cells was repeated four times; then, the lower fraction comprising the high-density cells was inoculated on S solid medium and cultured for four days at 30 °C.

### 2.6. Large-Scale Cultivation Condition

The *L. starkeyi* mutant cells with decreased lipid productivities than the wild-type strain were isolated and characterized under the culture conditions as previously described [25]. Briefly, the wild-type and mutant cells were cultured in 50 mL of SG medium in a 200 mL baffled flask at 30 °C for three days at 160 rpm. The resulting cultures were transferred to 300 mL of S medium (5% glucose) in a 500 mL baffled flask at 3.0 × 10^6^ cells/mL and precultured for one day at 30 °C and 160 rpm. Then, the precultured yeast cells with an initial cell density of 1.2 × 10^7^ cells/mL were inoculated into a 500 mL baffled flask with 300 mL of S medium (5% glucose) and cultivated at 30 °C and 160 rpm.

### 2.7. Small-Scale Cultivation Condition

*L. starkeyi* ∆*lslig4*, ∆*lslig4*∆*acl1*∆*acl2*, ∆*lslig4*/*ACL1ACL2,* sr22, and sr22/*ACL1ACL2* cells were cultured in 50 mL of S medium (5% glucose), with or without 0.05% acetate, in a 200 mL baffled flask for three days at 30 °C and 160 rpm. The cells cultured in the abovementioned medium were transferred to 75 mL of S medium, with or without 0.05% acetate, in a 200 mL baffled flask to yield a cell concentration of 1.25 × 10^6^ cells/mL and cultured at 30 °C for one day. The resultant culture was inoculated at an initial cell density of 1.25 × 10^6^ cells/mL in 75 mL of S medium, with or without 0.05% acetate, in a 200 mL baffled flask and cultivated at 30 °C.

### 2.8. Microscopy

Differential interference contrast (DIC) microscopy was performed using a BX53 microscope (Olympus, Tokyo, Japan).

### 2.9. Quantification of TAG Content

The extraction and quantification of the amount of intracellular lipids (mainly TAG) were carried out as previously described [24]. The cells harvested from the liquid culture were used to prepare a cell suspension (OD 660 = 20) with 500 µL of PBS in a 2 mL tube. The cells were broken by 1 g of glass beads (0.5 mm in diameter, AS ONE, Osaka, Japan) at 25 °C using a Multi-Beads Shocker (Yasui Kikai, Osaka, Japan) for 15 min at 2500 rpm. After that, 500 µL of PBS was added to a 2 mL tube and shaken using a Micro Mixer (TAITEC, Saitama, Japan). The determination of TAG and glycerol in the broken cell samples were enzymatically analyzed using TG E-test (FUJIFILM Wako Pure Chemical) and F-Kit glycerol (Roche Diagnostics, Tokyo, Japan), following the manufacturer’s instructions. Assuming that the total TAG was constituted by triolein, the amount of TAG was determined as the difference in the values obtained using the TG E-test and F-Kit for glycerol.

### 2.10. Quantification of the Glucose Concentration of the Culture Supernatant

The measurement of the glucose concentration was carried out as previously described [24]. The glucose concentration of the contained culture supernatant was measured using a Wako Glucose C-II Test (FUJIFILM Wako), following the manufacturer’s instruction.

### 2.11. Genomic DNA Extraction

The yeast cells (40 units of optical density at 660 nm) cultured with a YPD liquid medium were collected in a 2 mL tube via centrifugation at 10,000× *g* for 1 min at room temperature. The supernatant was discarded, and the cell pellet was washed twice with distilled water. The resultant cells were suspended with 250 µL of lysing buffer (100 mM NaCl (FUJIFILM Wako), 10 mM Tris-HCl (pH 8.0), and 1 mM EDTA (pH 8.0)), then, the cell suspension was mixed with 250 µL of TE-phenol (phenol equilibrated to TE )10 mM Tris-HCl, pH 8.0; 1 mM EDTA)), phenol-chloroform, and 1 g of glass beads (0.5 mm diameter, AS ONE). The cells were broken by vortexing using a Delta Mixer Se-08 (TAITEC) for 20 min at room temperature. The resultant mixture was mixed with 400 µL of TE-phenol. After the centrifugation at 18,000× *g* for 10 min at room temperature, the upper layer (250 µL) was transferred to a 1.5 mL test tube and vortexed with 250 µL of phenol-chloroform. After the centrifugation at 18,000× *g* for 10 min at room temperature, the upper layer (200 µL) was transferred to a 1.5 mL test tube. The supernatant was mixed with 20 µL of 3 M sodium acetate and 500 µL of ethanol. The mixture was allowed to stand for 5 min at room temperature; then, it was centrifugated at 18,000× *g* for 5 min at room temperature. Removing the supernatant, the resultant pellet was rinsed with 200 µL of 70% ethanol. After the centrifugation at 18,000× *g* for 2 min at room temperature, the supernatant was removed. The pellet was dissolved in a TE-RNaseA buffer (10 mM Tris-HCl (pH 8.0), 1 mM EDTA (pH 8.0), and 50 µg/mL RNaseA (Nippon Gene, Toyama, Japan)) and incubated at 37 °C for 2 h.

### 2.12. Quantitative Real Time PCR (qRT-PCR) Analysis

Total RNA extraction, reverse transcription, and quantitative real time polymerase chain reaction (qRT-PCR) analysis were done as previously described [25]. Briefly, the yeast cells harvested from 3 mL of liquid culture were washed with PBS. The resulting washed cells were suspended in 350 µL of TES buffer (10 mM Tris-HCl (pH 7.5), 10 mM ethylenediaminetetraacetic acid (pH 7.5), and 0.5% sodium lauryl sulfate) and 350 µL of water-saturated phenol in a 2 mL tube. The mixtures were disrupted with 1.4 g of zirconia beads (0.5 mm in diameter, YTZ-0.5; AS ONE) using a Multi-Beads Shocker at 2500 rpm for 12 cycles of 60 s of agitation and 60 s of rest at 4 °C. The homogenate was transferred to a 2 mL tube and centrifuged at 20,000× *g* for 10 min at 4 °C. The upper layer (350 µL) was transferred to a 1.5 mL tube and mixed with ISOGENII (Nippon Gene). After vortexing, the tube was set aside for 5 min at room temperature. The solution was vortexed with 240 µL of chloroform and centrifuged at 20,000× *g* for 5 min at room temperature. About 800 µL of the upper layer was transferred to a 1.5 mL test tube and mixed with 480 µL of ethanol. The mixture was further purified using an Illustra RNAspin Mini RNA Isolation Kit (Cytiva, Piscataway, NJ, USA), following the manufacturer’s instructions.

The first-strand cDNA was reverse transcribed from the extracted RNA using a PrimeScript RT reagent Kit (TaKaRa Bio, Shiga, Japan) following the manufacturer’s instructions.

Quantitative real time PCR analysis was performed using TB Green Premix Ex Taq II (TaKaRa Bio) on the Thermal Cycler Dice Real Time System III TP950 (TaKaRa Bio). The reaction mixture (20 µL) contained 10 µL of TB Green Premix Ex Taq II (Tli RNaseH Plus), 0.8 µL of 10 µM forward primer, 0.8 µL of 10 µM reverse primer, 2 µL of the DNA template, and 6.4 µL of sterile water. The primers for qRT-PCR were previously described by Takaku et al. [25] (Table S2). The PCR reaction conditions included an initial desaturation step at 95 °C for 30 s and 40 cycles of 95 °C for 5 s and 60 °C for 30 s. The final dissociation step was run at 95 °C for 15 s, 60 °C for 30 s, and 95 °C for 15 s. The relative changes in the transcriptional levels of genes were estimated using a relative standard curve method and normalized to the 18S rRNA gene. The standard curves for the relative quantification of target genes were obtained using the genomic DNA of wild-type *L. starkeyi*. For each gene, the standard genomic DNAs were amplified together with sample cDNAs in the same PCR run. The genomic DNA of *L. starkeyi* was prepared as mentioned above by genomic DNA extraction.

### 2.13. General Molecular Biology Techniques

The genomic DNA of *L. starkeyi* strains was prepared as mentioned above. The plasmid DNA was extracted using a FastGene^®^ Plasmid Mini Kit (Nippon Genetics Co., Ltd., Tokyo, Japan). PCR was performed using KOD One^®^ PCR Master Mix DNA polymerase in accordance to the manufacturer’s instructions (Toyobo Co., Ltd., Osaka, Japan). The amplified PCR products were extracted using a FastGene^®^ Gel/PCR extraction Kit (Nippon Genetics Co., Ltd.).

### 2.14. Construction of the Disruption Cassette Plasmid pKS-hph-ACL1-ACL2

To disrupt the *L. starkeyi* ACL genes (*ACL1* (gm1.5446_g) and *ACL2* (gm1.5447_g)), a pKS-hph-ACL1-ACL2 plasmid was constructed. It contained the *LsTDH3* promoter/hph/*LsTDH3* terminator DNA fragment for expression and the untranslated regions (UTRs) of the *ACL1* and *ACL2* genes. The plasmid pKS-hph-ACL1-ACL2 was assembled with four PCR fragments using NEBuilder^®^ HiFi DNA Assembly (New England BioLabs, Ipswich, MA, USA). The PCR fragments of 5′- and 3′-UTRs of the *ACL1* and *ACL2* genes were amplified with the primer sets ACL1ACL2-5′UTR-Fw/ACL1ACL2-5′UTR-Rv and ACL1ACL2-3′UTR-Fw/ACL1ACL2-3′UTR-Rv, respectively, using the genomic DNA of *L. starkeyi* CBS1807 as a template. The PCR fragment containing the *LsTDH3* promoter region, hygromycin B resistance marker (*hph*) and *LsTDH3* terminator region was amplified with the primer set ACL1ACL2-P+hph+T-Fw/ACL1ACL2-P+hph+T-Rv using the plasmid pKS-18S-hph [13]. The PCR fragment of the vector region was amplified with the primer set Vector-Fw/Vector-Not1-site-Rv using the plasmid pKS-18S-hph as a template. The PCR primers used for plasmid construction are described in Table 1. The DNA fragment used for yeast transformation was obtained by digesting the plasmid pKS-hph-ACL1-ACL2 with *Not*I (Takara Bio).

### 2.15. Construction of the High Expression Cassette Plasmid pKS-PTDH3-ACL1-sNAT1-PTDH3-ACL2

To express a high level of *ACL1* and *ACL2* genes, we constructed the plasmid pKS-PTDH3-ACL1-sNAT1-PTDH3-ACL2 containing the *LsTDH3* promoter/*ACL1*/*LsTDH3* terminator, *LsACT1* promoter/*sNAT1*/*LsACT1* terminator, and *LsTDH3* promoter/*ACL2*/*LsTDH3* terminator DNA fragments for the expression of *ACL1*, nourseothricin resistance marker (*sNAT1*), and *ACL2* genes, respectively, in *L. starkeyi* and the 5′- and 3′-UTRs of *LsLIG4* genes. The four DNA fragments, *LsLIG4* 3′UTR/Vector region/*LsLIG4* 5′UTR, *LsTDH3* promoter/*ACL1*/*LsTDH3* terminator, *LsACT1* promoter/*sNAT1*/*LsACT1* terminator, and *LsTDH3* promoter/*ACL2*/*LsTDH3* terminator, were assembled using NEBuilder^®^ HiFi DNA Assembly.

To construct the DNA fragment of *LsLIG4* 3′UTR/Vector region/*LsLIG4* 5′UTR, the DNA fragments of the 5′ and 3′ UTRs of the *LsLIG4* gene were amplified with the primer sets of LsLIG4-5′UTR-Fw2/LsLIG4-5′UTR-Rv and LsLIG4-3′UTR-Fw2/LsLIG4-3′UTR-Rv, respectively, using the genomic DNA of *L. starkeyi* CBS1807 as a template. The DNA fragment of the vector region was amplified with the primer set of Vector-Fw2/Vector-Rv, using the plasmid pKS-18S-hph [13] as a template. The DNA fragment of *LsLIG4* 3′UTR/Vector region/*LsLIG4* 5′UTR was amplified with a primer set of LsLIG4-3′UTR-Fw2/LsLIG4-5′UTR-Rv using the assembled DNA fragment (*LsLIG4* 3′UTR, Vector region, *LsLIG4* 5′UTR), with NEBuilder^®^ HiFi DNA Assembly as a template.

To construct the DNA fragment of *LsTDH3* promoter/*ACL1*/*LsTDH3* terminator region, the DNA fragments of *LsTDH3* promoter and *LsTDH3* terminator were amplified using the primer sets of ACL1-PTDH3-Fw/ACL1-PTDH3-Rv and ACL1-TTDH3-Fw/ACL1-TTDH3-Rv, respectively. The DNA fragment of *ACL1* was amplified with the primer set of ACL1-cDNA-Fw/ACL1-cDNA-Rv using the cDNA of *L. starkeyi* CBS1807 as a template. The DNA fragment of *LsTDH3* promoter/*ACL1*/*LsTDH3* terminator region was amplified with the primer set of ACL1-PTDH3-Fw/ACL1-TTDH3-Rv using the assembled DNA fragment (*LsTDH3* promoter, *ACL1*, and *LsTDH3* terminator region) as a template using NEBuilder^®^ HiFi DNA Assembly.

To construct the fragment of *LsACT1* promoter/*sNAT1*/*LsACT1* terminator region, the DNA fragments of *LsACT1* promoter and *LsACT1* terminator were amplified with the primer sets of PACT1-Fw/PACT1-Rv and TACT1-Fw/TACT1-Rv, respectively, using the genomic DNA of *L. starkeyi* CBS1807 as a template [27]. The DNA fragment of the *sNAT1* region was amplified with the primer set of sNAT1-PACT1-Fw/sNAT1-PACT1-Rv using pKS-LsLIG4-sNAT1 [15] as a template. Using NEBuilder^®^ HiFi DNA Assembly, the DNA fragment of *LsACT1* promoter/*sNAT1*/*LsACT1* terminator region was amplified using the primer set of PACT1-Fw/TACT1-Rv, with the assembled DNA fragment (*LsACT1* promoter, *sNAT1*, *LsACT1* terminator region) as a template.

Finally, to construct the DNA fragment of *LsTDH3* promoter/*ACL2*/*LsTDH3* terminator region, the DNA fragments of *LsTDH3* promoter and *LsTDH3* terminator were amplified with the primer sets of ACL2-PTDH3-Fw/ACL2-PTDH3-Rv and ACL2-TTDH3-Fw/ACL2-TTDH3-Rv, respectively, using the genomic DNA of *L. starkeyi* CBS1807 as a template. The DNA fragments of *ACL2* were amplified with the primer set of ACL2-cDNA-Fw/ACL2-cDNA-Rv using the cDNA of *L. starkeyi* CBS1807 as a template. The DNA fragment of *LsTDH3* promoter/*ACL2*/*LsTDH3* terminator region was amplified with the primer set of ACL2-TTDH3-Fw/ACL2-TTDH3-Rv using the assembled DNA fragment (*LsTDH3* promoter, *ACL2*, and *LsTDH3* terminator region) as a template by NEBuilder^®^ HiFi DNA Assembly. The PCR primers used for plasmid construction as described in Table 1. The DNA fragment used for yeast transformation was obtained by digesting the plasmid pKS-PTDH3-ACL1-sNAT1-PTDH3-ACL2 with ApaI (Takara Bio).

### 2.16. Yeast Transformation

The transformation of *L. starkeyi* by electroporation was performed as described previously [15]. Briefly, *L. starkeyi* ∆*lslig4* or sr22 cells were cultured in YPD to the mid-log phase (OD660, 6.0). The cells were put on ice and collected by centrifugation. After removing the supernatant, the cells were suspended with 8 mL of sterilized distilled water. After adding 1 mL of TE and 1 mL of 2 M LiAc, the cell suspension was incubated at 30 °C for 45 min with slow shaking. Then, 100 µL of 1 M dithiothreitol (DTT) was added, and the cell suspension was incubated at 30 °C for 15 min with slow shaking. After being diluted to 50 mL with ice-cold sterilized distilled water, the cells were harvested by centrifugation, washed with 50 mL of ice-cold sterilized distilled water, and resuspended with 3 mL of 0.5 M ice-cold sucrose. After the centrifugation, 40 µL of the resultant cell pellets and 1 µg of the DNA fragments for yeast transformation were mixed in a chilled 1.5 mL microtube, transferred to a chilled 0.2 cm electroporation cuvette (Bio-Rad Laboratories, Hercules, CA, USA), and pulsed with the conditions of capacitance at 25 µF, field strength at 3.75 kV/cm, and resistance at 800 Ω using the Gene Pulser X-Cell (Bio-Rad Laboratories). After pulsing, 1 mL of YPD medium with 0.5 M sucrose was immediately added to the cuvette, mixed gently, transferred into 4 mL of YPD medium with 0.5 M sucrose, and incubated at 30 °C for 10 h with slow shaking. When the culture was centrifugated, the resultant pellet was resuspended in sterilized distilled water, inoculated on YPD agar plate containing 100 µg/mL hygromycin B or 30 µg/mL nourseothricin, and incubated at 30 °C for four days. To confirm the target DNA integration in the transformants, PCR was performed using the obtained transformant colony as a template and the primer sets of ACL1-3′UTR-out-Fw/ACL1-3′UTR-out-Rv for *ACL1* and *ACL2* locus insertion or LsLIG4-5′UTR-out-Fw/LsLIG4-3′UTR-out-Rv for *LsLIG4* locus insertion (Figure S1A,B,D,E,G,H). We also confirmed the target DNA integration by Southern blot analysis of the genomic DNA of the transformants (Figure S1A,C,D,F,G,I).

### 2.17. Statistical Analysis

The statistical analysis was performed using *t*-test in GraphPad Prism software, version 8 (GraphPad Software Inc., San Diego, CA, USA). The differences were considered significant at *p* < 0.05.

## 3. Results

### 3.1. Screening of Mutants with Decreased Lipid Productivity Than Wild-Type L. Starkeyi Strain

**Figure 1:**
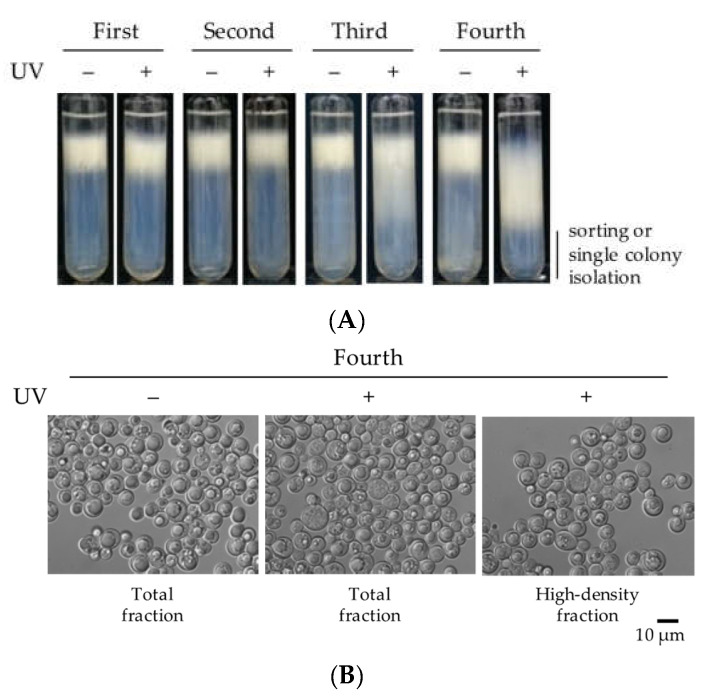
Isolation of the mutant strain sr22 in the screening. (**A**) Enrichment of high-density cells by Percoll density gradient centrifugation four times after UV irradiation (+) or not (−). The cells cultured for six days on S medium were separated by Percoll density gradient centrifugation (90%). The bottoms (3 mL) of the sample fractions after the centrifugation were subjected to sorting or single colony isolation. (**B**) Differential interference contrast (DIC) microscopy images of UV-untreated cells in the total fraction and UV-treated cells in the total and high-density fractions.

To obtain the mutants with decreased TAG productivity than the wild-type *L. starkeyi* strain, the mutagenesis treatment of wild-type cells by UV irradiation was done as described in Materials and Methods. The cell viability rate of UV-treated cells was 3.9%. We previously obtained *L. starkeyi* mutants with increased lipid productivity using Percoll density gradient centrifugation [24,25]. This procedure could be applied to the enrichment of high-density cells that were expected to accumulate less TAGs. In this study, we attempted to enrich the high-density cells that were expected to lose the property of lipid accumulation. In the first level of screening, we performed the enrichment of high-density cells after six days of cultivation using Percoll density gradient centrifugation four times (Figure 1A). The microscopic observation of high-density fractions after the centrifugation of the fourth enrichment revealed a large proportion of cells with small lipid droplets in the UV-treated fraction (Figure 1B). After the single colony isolation on an S solid medium, 25 colonies were randomly selected from an S solid medium. In the second level of screening, the mutants with decreased lipid productivity than the wild-type strain were selected; the 25 strains picked were screened for lipid accumulation by microscopic observation after six days on an S medium culture. We measured the diameters of intracellular lipid droplets in 50 randomly selected cells in each strain and counted the cells with <2 µm lipid droplet diameter. The proportion of cells with <2 µm lipid droplet diameter was only 2% in the wild-type strain. All randomly selected cells in seven strains, No. 10–12, 16, 21, 22, and 24, had lipid droplet diameters <2 µm (Table S3). There was no significant difference in the degree/type of lipid accumulation, cell concentration, and cell size of the seven mutant strains on days 1 and/or 6 (Figure S2A–C). Therefore, these strains were expected to have similar cell growth and lipid accumulation capacities. We picked up No. 22, which had a slightly higher cell concentration than the other strains on day 6 and named it sr22.

### 3.2. Characterization of the Cell Growth and TAG Productivity of the sr22 Mutant

**Figure 2:**
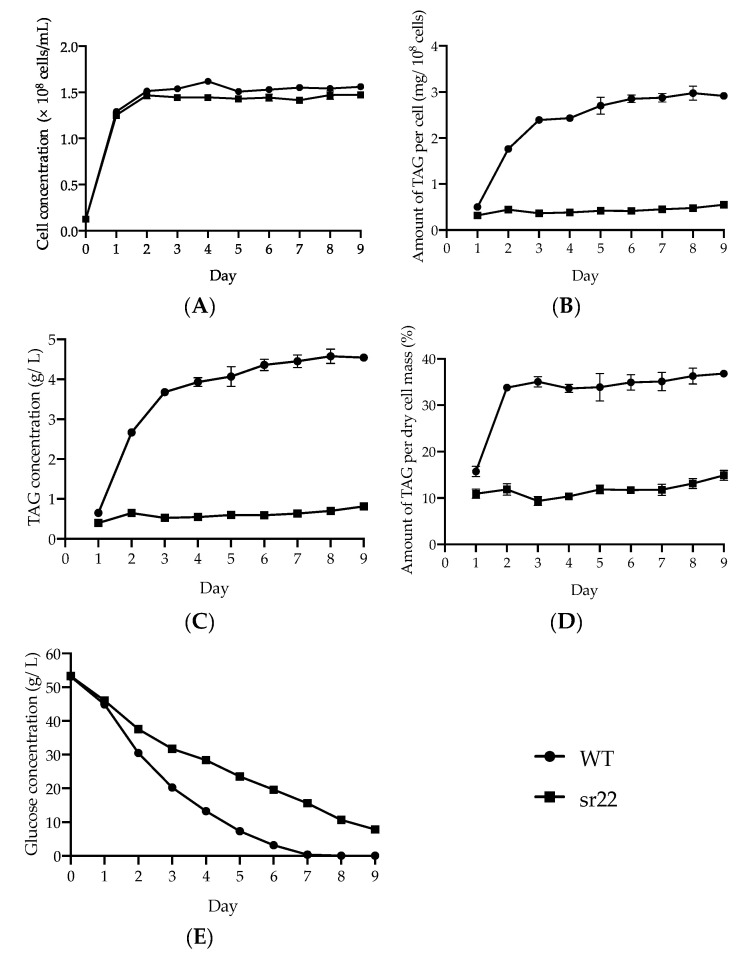
Characterization of the wild-type (WT) and mutant strain sr22. The WT and sr22 cells were cultured on an S medium at 30 °C for nine days in a large-scale culture. Symbols: closed circles, WT; closed squares, sr22. (**A**) Cell concentration (cells/mL), (**B**) amount of TAG per cell (mg/10^8^ cells), (**C**) TAG concentration (g/L), (**D**) amount of TAG per dry cell mass (%), and (**E**) glucose concentration (g/L). The data are indicated as the mean ± SEM of the three independent experiments.

To evaluate the cell growth and TAG production of the sr22 mutant in comparison with the wild-type strain over time, we cultured the wild-type and sr22 mutant cells on an S medium for nine days. The cell concentration of the sr22 mutant was slightly lower than that of the wild-type strain after day 2 (Figure 2A). After day 2, the TAG level per sr22 mutant cell was consistently lower than that of the wild-type strain, even though the TAG levels in the wild-type strain increased exponentially (Figure 2B). Similarly, in the sr22 mutant, the size of intracellular lipid droplets observed in a microscope remained significantly smaller than that of the wild-type strain after day 2 (Figure S3). The TAG concentration in the sr22 mutant was consistently lower than that of the wild-type strain; that of the sr22 mutant was only 15–20% of that of the wild-type strain after day 3 (Figure 2C). The TAG level per dry cell mass of the wild-type strain was >33% after day 2, whereas that of the sr22 mutant was only approximately 10% after day 1 (Figure 2D). The rate of glucose consumption of the sr22 mutant was slower than that of the wild-type strain after day 2, but it continued throughout the culture period (Figure 2E). On the other hand, the TAG level in the sr22 mutant did not increase throughout the culture period (Figure 2B–E). These results suggested that glucose was mainly converted into substances other than TAG in the sr22 mutant. While the glucose to TAG conversion yield of the wild-type strain was 11.2%, that of the mutant sr22 was only 2.4% on day 3 (Figure 2C,E). These results suggested that the decreased TAG productivity of the sr22 mutant was caused by the reduction in glucose consumption rate and decrease in the glucose to TAG conversion yield compared with the wild-type strain.

### 3.3. Comparison of the Transcription Expression Patterns of Citrate-Mediated Acyl-CoA Synthesis Pathway-Related Genes in the Wild-Type and sr22 Mutant

**Figure 3:**
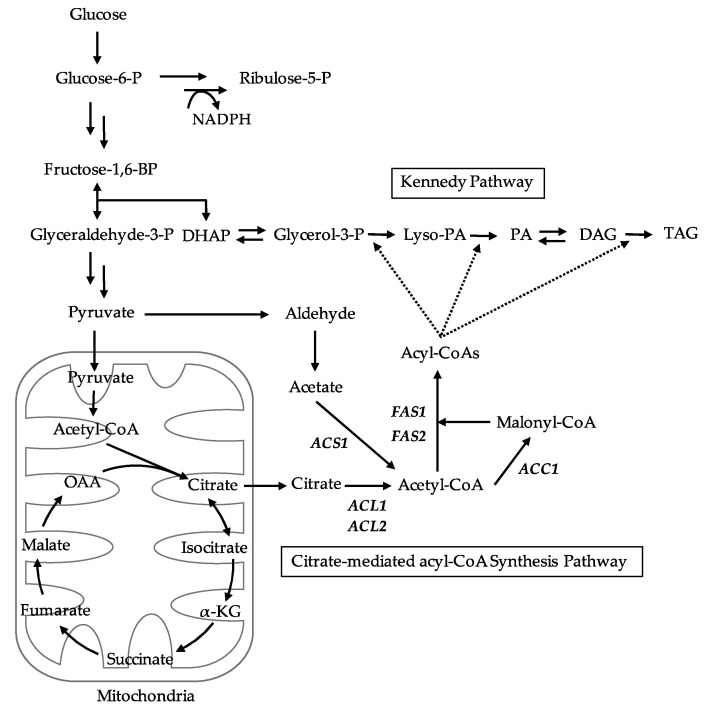
Overview of the principal metabolic pathways for TAG synthesis in *L. starkeyi.* Abbreviations for genes and metabolites are indicated as follows: *ACC1*, acetyl-CoA carboxylase; *ACL1*/*ACL2*, ATP-citrate lyase; *ACS1*, acetyl-CoA synthetase; α-KG, α-ketoglutarate; DAG, diacylglycerol; DHAP, dihydroxyacetone phosphate; *FAS1*/*FAS2*, fatty acid synthase subunit; Fructose-1,6-BP, fructose-1,6-bisphosphatase; Glucose-6-P, glucose-6-phosphate; Glyceraldehyde-3-P, glyceraldehyde-3-phosphate; Glycerol-3-P, glycerol-3-phosphate; Lyso-PA, lysophosphatidic acid; NADPH, reduced nicotinamide adenine dinucleotide phosphate; OAA, oxaloacetate; PA, phosphatidic acid; Ribulose-5-P, ribulose-5-phosphate; TAG, triacylglycerol.

In oleaginous yeasts, the citrate-mediated acyl-CoA synthesis pathway and Kennedy pathway play key roles in TAG synthesis [28]. After the synthesis of citrate by TCA cycle in the mitochondria, it is exported to the cytosol and converted to acetyl-CoA by ACL [29,30]. Acetyl-CoA carboxylase (ACC1) catalyzes the conversion of malonyl-CoA from acetyl-CoA [31]; then, the fatty acid synthase (FAS1, FAS2) complex synthesizes long-chain acyl-CoA from acetyl-CoA and malonyl-CoA using NADPH for reduction [32,33] (citrate-mediated acyl-CoA synthesis pathway; Figure 3). In our previous studies, the citrate-mediated acyl-CoA synthesis pathway-related genes greatly increased lipid productivity; mutants were expressed higher than in the parental strains on day 1 and 3. These results suggested that the high expression levels of citrate-mediated acyl-CoA synthesis pathway-related genes are important for TAG synthesis in *L. starkeyi* [25]. To further understand the importance of citrate-mediated acyl-CoA synthesis pathway on TAG synthesis, the sr22 mutant and WT cells were cultured in an S medium for three days (Figure S4), and the expression of citrate-mediated acyl-CoA pathway-related genes was investigated by qRT-PCR analysis.

**Figure 4:**
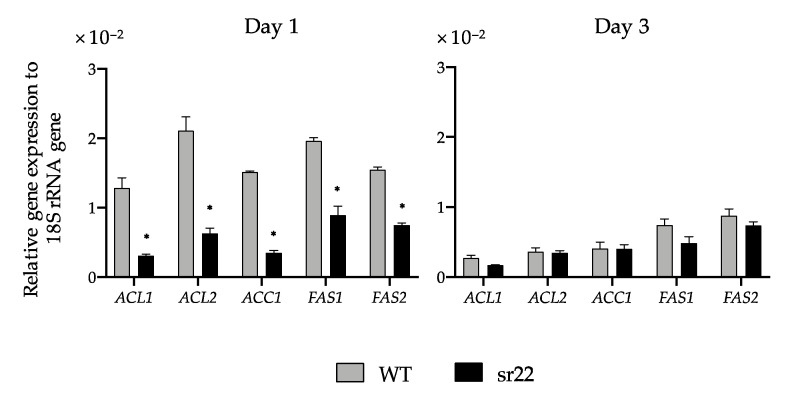
Relative transcription levels for citrate-mediated acyl-CoA synthesis pathway of sr22 compared with the wild-type strain (WT). WT and sr22 were cultured on an S medium at 30 °C for nine days in a large-scale culture. Symbols: gray bars, WT; black bars, sr22. The transcription levels with relative quantitative method normalized by 18S rRNA. The data are indicated as the mean ± SEM of the three independent experiments. Asterisks indicate statistically significant differences between WT and sr22 (*t*-test; * *p* < 0.05).

The expression levels of citrate-mediated acyl-CoA synthesis pathway-related genes (*ACL1*, *ACL2*, *ACC1*, *FAS1*, and *FAS2*) of the sr22 mutant were all downregulated compared with those of the WT strain on day 1. On day 3, those of the sr22 mutant were similar to those of the WT strain and were lower than those on day 1 (Figure 4). These results imply that the low expression of citrate-mediated acyl-CoA synthesis pathway-related genes in the sr22 mutant as compared with the WT strain at the early stage of culture (day 1) may lead to a decrease in TAG productivity.

### 3.4. Deletion of ACL1 and ACL2 Genes Coding for ATP-Citrate Lyase Altered the Growth and TAG Synthesis

**Figure 5:**
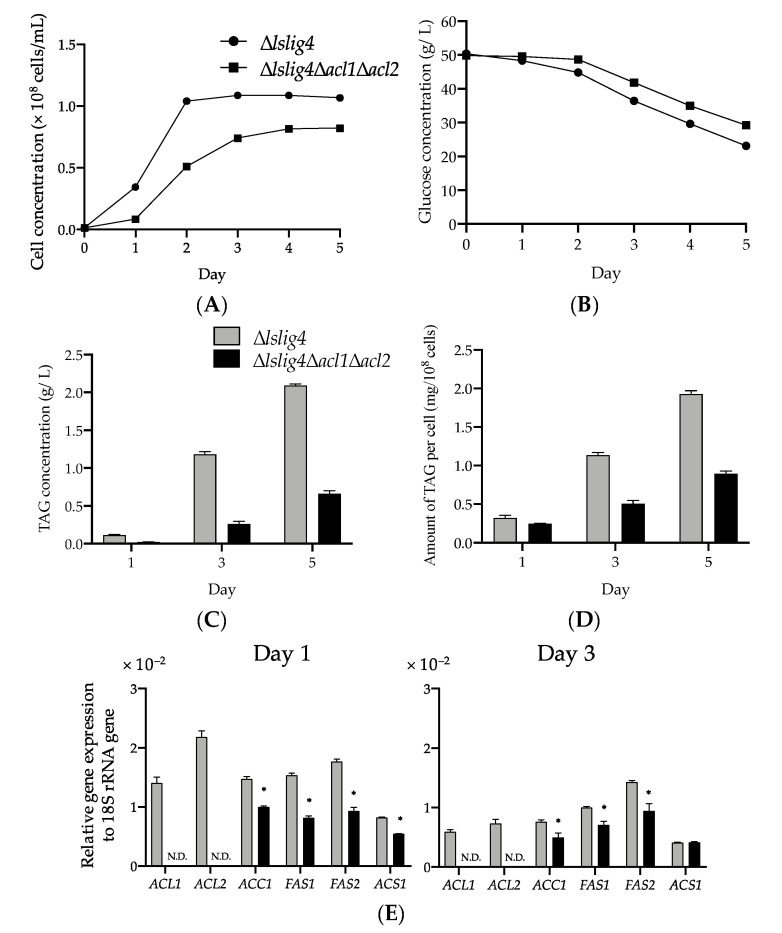
The effect of ATP-citrate lyase deletion on TAG synthesis in an S medium containing glucose. ∆*lslig4* and ∆*lslig4*∆*acl1*∆*acl2* were cultured in an S medium containing glucose at 30 °C for five days in a small-scale culture. Symbols: closed circles, ∆*lslig4*; closed squares, ∆*lslig4*∆*acl1*∆*acl2*; gray bars, ∆*lslig4*; black bars, ∆*lslig4*∆*acl1*∆*acl2*. N.D. indicates not detected. (**A**) Cell concentration (cells/mL), (**B**) glucose concentration (g/L), (**C**) TAG concentration (g/L), (**D**) amount of TAG per cell (mg/10^8^ cells), and (**E**) relative expression levels of genes involved in acyl-CoA synthesis (*ACL1*, *ACL2*, *ACC1*, *FAS1*, *FAS2*, and *ACS1*). The transcription levels with relative quantitative method normalized by 18S rRNA. The data are indicated as the mean ± SEM of the three independent experiments. Asterisks indicate statistically significant differences between ∆*lslig4* and ∆*lslig4*∆*acl1*∆*acl2* (*t*-test; * *p* < 0.05).

To further clarify the importance of citrate-mediated acyl-CoA synthesis pathway on TAG synthesis, we constructed an *L. starkeyi* strain, ∆*lslig4*∆*acl1*∆*acl2,* in which the *ACL1* and *ACL2* genes coding for ACL were deleted, and we evaluated its TAG productivity. We observed that the growth, glucose consumption, TAG concentration, and TAG level per cell of ∆*lslig4*∆*acl1*∆*acl2* were reduced compared with those of the reference strain (∆*lslig4*) (Figure 5A–D). The cell concentration of ∆*lslig4*∆*acl1*∆*acl2* was only 25%, 68%, and 77% compared with that of ∆*lslig4* on day 1, 3, and 5, respectively (Figure 5A). The amount of TAG per cell in ∆*lslig4*∆*acl1*∆*acl2* reached only 45% and 46% compared with that of ∆*lslig4* on day 3 and 5, respectively (Figure 5D). While the conversion rate of glucose to TAG in ∆*lslig4* was 8.5%, that in ∆*lslig4*∆*acl1*∆*acl2* was only 3.2% on day 3 (Figure 5B,C).

The two potential sources of cytosolic acetyl-CoA for acyl-CoA synthesis were acetate and citrate (Figure 3). The cytosolic acetyl-CoA in nonoleaginous yeast *S. cerevisiae* was converted from acetate via acetyl-CoA synthase, whereas that in oleaginous yeast *Y. lipolytica* was converted mainly from citrate via ACL [30,34]. To better understand the acyl-CoA synthesis in *L. starkeyi*, we investigated the expression of *ACS1*, *ACL1*, *ACL2, ACC1, FAS1*, and *FAS2* genes in ∆*lslig4* and ∆*lslig4*∆*acl1*∆*acl2* on day 1 and 3 (Figure 5E). The *ACL1* and *ACL2* genes were expressed in ∆*lslig4* but not in ∆*lslig4*∆*acl1*∆*acl2* on day 1 and 3. The expression levels of *ACC1, FAS1*, and *FAS2* genes in ∆*lslig4*∆*acl1*∆*acl2* were decreased in comparison with those in ∆*lslig4* on day 1 and 3. These results suggested that the upregulation of citrate-mediated acyl-CoA synthesis-related genes was required for growth and TAG accumulation at an early stage in a medium containing glucose.

**Figure 6:**
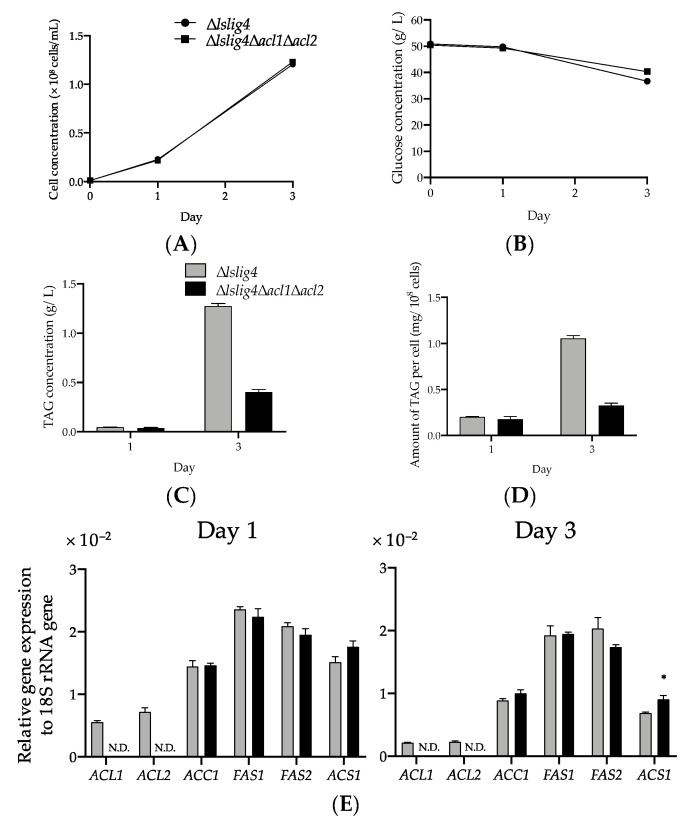
The effect of ATP-citrate lyase deletion on TAG synthesis on an S medium containing glucose and acetate. Symbols: closed circles, ∆*lslig4*; closed squares, ∆*lslig4*∆*acl1*∆*acl2*; gray bars, ∆*lslig4*; black bars, ∆*lslig4*∆*acl1*∆*acl2*. N.D. indicates not detected. (**A**–**E**) The analysis of cell concentration, glucose concentration, TAG concentration, amount of TAG per cell, and relative expression levels of genes involved in acyl-CoA synthesis were performed as described in Figure 5, except that the S medium containing glucose was replaced with an S medium containing glucose and acetate. Asterisks indicate statistically significant differences between ∆*lslig4* and ∆*lslig4*∆*acl1*∆*acl2* (*t*-test; * *p* < 0.05).

In oleaginous yeast *Cryptococcus neoformans*, the mutant lacking the *ACL1* gene that codes for ACL exhibited growth defects on a medium containing glucose as the only carbon source. Conversely, the growth of the *ACL1* deletion mutant was similar to that of the WT strain on an acetate medium, causing the disturbance in the glucose to cytosolic acetyl-CoA pathway and functional conversion of exogenous acetate to cytosolic acetyl-CoA by ACS1 in the *acl1* deletion mutant [35]. In accordance with this report, the ∆*lslig4* and ∆*lslig4*∆*acl1*∆*acl2* cells were cultured on an S medium containing glucose and acetate. Both strains showed that the similar growth rate (Figure 6A) and expression levels of *ACS1* in those strains cultured on the S medium containing glucose and acetate were increased compared with those on the S medium containing glucose (Figure 5E and Figure 6E). These results indicated the functional conversion of exogenous acetate to cytosolic acetyl-CoA by ACS1 in the *acl1* and *acl2* deletion mutant.

To further understand the importance of citrate-mediated acyl-CoA synthesis pathway on TAG synthesis, we analyzed the TAG level and expression of acyl-CoA synthesis-related genes of ∆*lslig4* and ∆*lslig4*∆*acl1*∆*acl2* in the culture medium containing glucose and acetate. The results showed that despite the recovering growth and expression levels of *ACC1*, *FAS1*, and *FAS2* due to the presence of acetate (Figure 5A,E and Figure 6A,E), the TAG productivity remained low as in the case of glucose medium culture in ∆*lslig4*∆*acl1*∆*acl2* (Figure 6B–D). This result suggested that the citrate-mediated-acyl-CoA synthesis pathway is very important for effective TAG synthesis.

We also constructed a strain highly expressing the *ACL1* and *ACL2* genes (∆*lslig4*/*ACL1ACL2*) and estimated the TAG productivity and expression of citrate-mediated acyl-CoA synthesis-related genes of those cells cultured in an S medium. The TAG productivity in ∆*lslig4*/*ACL1ACL2* was similar to those in ∆*lslig4* (Figure S5A). The expression levels of *ACL1* and *ACL2*, but not *ACC1*, *FAS1*, and *FAS2* of ∆*lslig4*/*ACL1ACL2*, were significantly increased compared with those of ∆*lslig4* (Figure S5B). These results indicated that the increased expression of *ACL1* and *ACL2* genes alone did not improve the TAG productivity.

## 4. Discussion

In this study, we obtained an *L. starkeyi* mutant, sr22, with significantly decreased lipid productivity. The sr22 mutant had decreased expression levels of acyl-CoA synthesis pathway-related genes (*ACL1*, *ACL2*, *ACC1*, *FAS1*, and *FAS2*) compared with those of the WT strain. We also revealed that the deletion of *ACL1* and *ACL2* genes coding for ACL, which is responsible for the first reaction of citrate-mediated acyl-CoA synthesis pathway, led to poor growth and decreased lipid productivity in the glucose medium.

Several mutants with increased lipid productivities were obtained in oleaginous yeasts, such as *R. glutinis* [17], *R. toruloides* [18,19,20], *Y. lipolytica* [21,22], *L. starkeyi* [23,24,25], and *C. curvatus* [26]. Some of these mutants have enhanced lipid productivities and increased expression of acyl-CoA synthesis-related genes compared with their parent strains [19,20,25,26,36]. However, to our knowledge, there are no recent studies that obtained mutants with greatly decreased lipid productivity and performed the expression analysis of TAG synthesis-related genes in oleaginous yeasts. The isolation and analysis of mutants with increased lipid productivities and decreased lipid productivities will potentially provide valuable information on TAG synthesis. We previously established a significantly efficient method to enrich the low-density cells, which produced an increased amount of TAG. In this study, given that the decrease in TAG in cells corresponds to the increase in intracellular density, we tried to efficiently isolate the mutants with decreased lipid productivities using the highly selective screening strategy in reverse to enrich the high-density cells, which were expected to accumulate less TAG [24]. The cells with a different amount of TAG were efficiently fractionated and enriched by Percoll density gradient centrifugation. This method is expected to be widely used to isolate TAG accumulation in cells based on their intracellular densities in oleaginous microorganisms.

The expression levels of citrate-mediated acyl-CoA synthesis pathway-related genes (*ACL1*, *ACL2*, *ACC1*, *FAS1*, and *FAS2*) of the sr22 mutant with greatly decreased lipid productivity were significantly lower than those of the WT strain (Figure 4). We previously exhibited the significantly increased expression of *ACL1*, *ACL2*, *ACC1*, *FAS1*, and *FAS2* genes in the *L. starkeyi* mutants with increased lipid productivities (E15, E15-11, E15-15, and E15-25) [25]. In some oleaginous yeast mutants, the elevated levels of acyl-CoA synthesis pathway-related gene expression were reported to lead to improved lipid productivity. The expressions of *ACL1*, *FAS1*, and *FAS2* genes in the *R. toruloides* mutants R-ZY13 and 8766 3-11C with increased lipid productivities exceeded those in the parent strains [20,26]. In *Y. lipolytica*, the comparative transcriptomic analysis between the control strain and mutant E26 with significantly improved lipid productivity showed upregulated acyl-CoA synthesis-related genes in the mutant [36]. Hence, in oleaginous yeasts, the expression changes in *ACL1*, *ACL2*, *ACC1*, *FAS1*, and *FAS2* genes linked to the increase or decrease in TAG productivities and the overexpression of citrate-mediated acyl-CoA synthesis-related enzymes may be a driving force for lipogenesis.

We also showed that the ∆*lslig4*/*ACL1ACL2* strain with highly expressed *ACL1* and *ACL2* genes in the citrate-mediated acyl-CoA synthesis pathway could not enhance the TAG productivity as compared with ∆*lslig4* (Figure S5A). There was no difference in the expression levels of *ACC1*, *FAS1*, and *FAS2* genes between ∆*lslig4*/*ACL1ACL2* and the reference strain (∆*lslig4*) (Figure S5B), while the expression levels of *ACL1*, *ACL2*, *ACC1*, *FAS1*, and *FAS2* genes in *L. starkeyi* mutants with increased lipid productivities were largely increased as compared with those of the reference strain (wild-type). Furthermore, high expression of *ACL1* and *ACL2* genes in the sr22 mutant with greatly decreased lipid productivity did not restore the TAG productivity of sr22 mutant and had no effect on the expression levels of *ACC1*, *FAS1*, and *FAS2* genes of sr22 mutant (Figure S6A,B). Thus, we propose that the enhancement of TAG productivity is required for the upregulation of *ACL1*, *ACL2*, *ACC1*, *FAS1*, and *FAS2* genes in *L. starkeyi*.

∆*lslig4*∆*acl1*∆*acl2* exhibited poor cell growth, slow glucose consumption rate, and low TAG accumulation in the S medium containing glucose compared with the reference strain (∆*lslig4*) (Figure 5A–D). ∆*lslig4*∆*acl1*∆*acl2* also exhibited decreased expressions of *ACS1*, *ACC1*, *FAS1*, and *FAS2* genes in the S medium containing glucose compared ∆*lslig4* (Figure 5E). The growth and the expression levels of *ACS1*, *ACC1*, *FAS1*, and *FAS2* genes in ∆*lslig4*∆*acl1*∆*acl2* were recovered to the same level as those of the reference strain ∆*lslig4* when acetate was added to the S medium containing glucose (Figure 6A,E). However, the TAG productivity in ∆*lslig4*∆*acl1*∆*acl2* remained lower than that of the reference strain ∆*lslig4* (Figure 6B–D). In conclusion, our results suggest that cytosolic acyl-CoA is required for the growth and TAG accumulation of *L. starkeyi*. Especially, the supply of citrate-derived cytosolic acyl-CoA produced via citrate-mediated synthesis is responsible for its high TAG accumulation and growth at an early stage. The promotion of TAG synthesis requires the upregulation of citrate-mediated acyl-CoA synthesis-related genes (*ACL1*, *ACL2*, *ACC1*, *FAS1*, and *FAS2*). This study will help us increase the TAG accumulation and expand our current knowledge on the regulation of TAG synthesis in *L. starkeyi*. Our future research will improve the TAG productivity by a simultaneous high expression of citrate-mediated acyl-CoA synthesis-related genes by genetic engineering.

## Figures and Tables

**Table 1:** List of primers and primer sequences used for plasmid construction in this study.

Primer	Primer Sequence (5′–3′)	Reference
ACL1ACL2-5′UTR-Fw	TCAGCAAATTAAATAGTTTGTTGTGATATGTTCGCTGG	This study
ACL1ACL2-5′UTR-Rv	CACAACAAACTATTTAATTTGCTGAAGCGGTTTGCC	This study
ACL1ACL2-3′UTR-Fw	CACCCGCTACATGCGCTGGGCGCTTATTATG	This study
ACL1ACL2-3′UTR-Rv	GGGCTGCAGGAATTCGATGGGGCCCCTCTGAACATTCAAAATGGCGG	This study
ACL1ACL2-P+hph+T-Fw	CACAACAAACTATTTAATTTGCTGAAGCGGTTTGCC	This study
ACL1ACL2-P+hph+T-Rv	AAGCGCCCAGCGCATGTAGCGGGTGGTGATG	This study
Vector-Fw	GGGCCCCATCGAATTCCTGC	This study
Vector-Not1-site-Rv	GCGGCCGCGGGCCCATCAAGCTTATCGATAC	This study
LsLIG4-5′UTR-Fw2	CGGTATCGATAAGCTTGATGGGCCCTCGACGTATAACATACAAATAGCC	This study
LsLIG4-5′UTR-Rv	AGCAAATTAATGAGTTACCACAATTATATGCACATGGAGTC	This study
LsLIG4-3′UTR-Fw2	TGTTCCACCATCAGCCTGTTATAGAAGTCAAGTTCGC	This study
LsLIG4-3′UTR-Rv	GCAGGAATTCGATGGGGCCCGCGAGGATCATACATATCCTCAC	This study
Vector-Fw2	GGGCCCCATCGAATTCCTGC	This study
Vector-Rv	GGGCCCATCAAGCTTATCG	This study
ACL1-P_TDH3_-Fw	AATTGTGGTAACTCATTAATTTGCTGAAGCGGTTTGC	This study
ACL1-P_TDH3_-Rv	AGGAGGAGACATTGCGAATGTGGATTAGAGTAAGATAGATAAC	This study
ACL1-cDNA-Fw	ATCCACATTCGCAATGTCTCCTCCTTCTGCC	This study
ACL1-cDNA-Rv	ATCAACCGCACACCTAGACCGTGACTTCAACGC	This study
ACL1-T_TDH3_-Fw	GTCACGGTCTAGGTGTGCGGTTGATGGTCTTC	This study
ACL1-T_TDH3_-Rv	GGTAGACGGTAATGATGGTGGAACAAAGTTGTTTTTAAGATC	This study
P_ACT1_-Fw	TGTTCCACCATCATTACCGTCTACCGCTGACG	This study
P_ACT1_-Rv	AGTGGTACCCATTGTGAAATATTATACAATTAACTGTAGAAAGACAAAAATGGG	This study
sNAT1-P_ACT1_-Fw	ATAATATTTCACAATGGGTACCACTCTTGACGAC	This study
sNAT1-T_ACT1-_Rv	GAACAACGTCCGCTTAGGGGCAGGGCATGCTC	This study
T_ACT1_-Fw	CCCTGCCCCTAAGCGGACGTTGTTCCGGATG	This study
T_ACT1_-Rv	CAGCAAATTAATGAGGAGTATAGAGTTGAATTTAATGGACGTTG	This study
ACL2-P_TDH3_-Fw	CTCTATACTCCTCATTAATTTGCTGAAGCGGTTTGC	This study
ACL2-P_TDH3_-Rv	CTTTGCGGACATTGCGAATGTGGATTAGAGTAAGATAGATAAC	This study
ACL2-cDNA-Fw	ATCCACATTCGCAATGTCCGCAAAGTCCATCC	This study
ACL2-cDNA-Rv	ATCAACCGCACACTTACAGCACACTGACGGC	This study
ACL2-T_TDH3_-Fw	AGTGTGCTGTAAGTGTGCGGTTGATGGTCTTC	This study
ACL2-T_TDH3_-Rv	TCTATAACAGGCTGATGGTGGAACAAAGTTGTTTTTAAGATC	This study

## Data Availability

Data supporting results can be found in Supplementary Figures S1–S6 and Tables S1–S3.

## Supplementary Materials

The following are available online at https://www.mdpi.com/article/10.3390/microorganisms9081693/s1, Figure S1: PCR and Southern blot confirmations of *ACL1* and *ACL2* deletion or high expression, Figure S2: Phenotypes of mutants with greatly decreased lipid productivity strains (No. 10, 11, 12, 16, 21, 22, 24), Figure S3: Substantial differences in the DIC images of lipid droplets between the wild-type (WT) strain and sr22 mutant, Figure S4: Time course analysis of cell concentration, glucose concentration, amount of TAG per cell, and TAG concentration in the WT strain and sr22 mutant, Figure S5: The effect of ATP-citrate lyase overexpression on TAG synthesis on an S medium containing glucose, Figure S6: The effect of ATP-citrate lyase overexpression on TAG synthesis on an S medium containing glucose in sr22 mutant, Table S1: Microbial strains used in this study, Table S2: List of primers and primer sequences used for quantitative real time PCR and Southern blot analysis in this study, Table S3: Comparison of the lipid droplet diameter of the WT and mutants in the screening.
