# Supplementary material for: Citrate-Mediated Acyl-CoA Synthesis Is Required for the Promotion of Growth and Triacylglycerol Production in Oleaginous Yeast Lipomyces starkeyi
Source: Microorganisms. 2021 Aug 9;9(8):1693. doi: 10.3390/microorganisms9081693 (PMC8400019; doi:10.3390/microorganisms9081693)
Supplement: Supplementary file 1 [file microorganisms-09-01693-s001.zip › microorganisms-1333121-supplementary.pdf]

Supplementary data

Citrate-Mediated Acyl-CoA Synthesis Is Required for the Promotion of Growth and Triacylglycerol Production in Oleaginous Yeast *Lipomyces Starkeyi*

Rikako Sato [1], Satoshi Ara [1], Harutake Yamazaki [1], Koji Ishiya [2], Sachiyo Aburatani [3,*] and Hiroaki Takaku [1,*]

[1] Department of Applied Life Sciences, Niigata University of Pharmacy and Applied Life Sciences, Niigata 956-8603, Japan; sato.rikako03@gmail.com (R.S.); ara@nupals.ac.jp (S.A.); hyamazaki@nupals.ac.jp (H.Y.)

[2] Bioproduction Research Institute (BPRI), National Institute of Advance Industrial Science and Technology (AIST), Sapporo 062-8517, Japan; koji.ishiya@aist.go.jp

[3] Computational Bio Big-Data Open Innovation Laboratory (CBBD-OIL), National Institute of Advanced Industrial Science and Technology (AIST), Tokyo 135-0064, Japan

* Correspondence: s.aburatani@aist.go.jp (S.A.); htakaku@nupals.ac.jp (H.T.); Tel.: +81-3-35998712 (S.A.); +81-250-255119 (H.T.)

Supplementary Table

Table S1. Microbial strains used in this study

Strain name	Relevant genotype	Parental strain	Source of reference
Lipomyces starkeyi CBS1807			Centraalbureau voor Schimmelcultures
sr22		CBS1807	This study
Δ*lslig4*	Δ*lslig4*::P$_{TDH3}$-*Sh ble*-T$_{TDH3}$	CBS1807	[16]
Δ*lslig4*Δ*acl1*Δ*acl2*	Δ*lslig4*::P$_{TDH3}$-*Sh ble*-T$_{TDH3}$ Δ*acl1*Δ*acl2*:: P$_{TDH3}$-*hph*-T$_{TDH3}$	Δ*lslig4*	This study
Δ*lslig4*/*ACL1ACL2*	Δ*lslig4*::P$_{TDH3}$-*ACL1*-T$_{TDH3}$-P$_{ACT1}$-*sNAT1*-T$_{ACT1}$-P$_{TDH3}$-*ACL2*-T$_{TDH3}$	Δ*lslig4*	This study
sr22/*ACL1ACL2*	Δ*lslig4*::P$_{TDH3}$-*ACL1*-T$_{TDH3}$-P$_{ACT1}$-*sNAT1*-T$_{ACT1}$-P$_{TDH3}$-*ACL2*-T$_{TDH3}$	sr22	This study
Escherichia coli HST08	F −, endA1, supE44, thi-1, recA1, relA1, gyrA96, phoA, Φ80dlacZΔM15, Δ(lacZYA-argF)U169, Δ(mrr-hsdRMSmcrBC), ΔmcrA, λ−		TaKaRa Bio

Table S2. List of primers and primer sequences used for quantitative real time PCR and southern bot analysis in this study

Primer	Primer Sequence (5′–3′)	Reference
ACL1 Fw	GCCAAGAATGCCGCCATGAA	[25]
ACL1 Rv	GCTCGGGCTTCGGAACAATG	[25]
ACL2 Fw	GTGGTATCGCGAACTTCACACAG	[25]
ACL2 Rv	GGTGGAGTTGGGACTGGTAGTC	[25]
FAS1 Fw	TCAACTGGGAGCAAGCCACA	[25]
FAS1 Rv	TGACACGGACACCAGTTCCC	[25]
FAS2 Fw	GATGGAGCCTGTTTTCGCGG	[25]
FAS2 Rv	GCGAATCTCGGACGTCTGGA	[25]
ACS1 Fw	ACTACTGACGAAGGTCGACGTG	This study
ACS1 Rv	ACATCGGGACAGTTCTTCAGGG	This study
18S rRNA Fw	GGTAATCTTGTGAAACTCCGTCGTG	[25]
18S rRNA Rv	CAAGCTGATGACTTGCGCTTACTAG	[25]
ACL1ACL2-probe-Fw2	CCCATGTTCGTCATGTTG	This study

ACL1ACL2-probe-Rv2	TTCGATGACTGAGCGATC	This study
LsLIG4-5′UTR-out-Fw	CTGGCCATCAGACAAGTG	This study
LsLIG4-3′UTR-out-Rv	GAGTATCCACGCTCACATC	This study
LsLIG4-5′UTR-probe-Fw	CCCATGTTCGTCATGTTG	This study
LsLIG4-5′UTR-probe-Rv	TTCGATGACTGAGCGATC	This study

Table S3. Comparison of the lipid droplet diameter of wild-type and mutants in the screening

Strain name and No	The counted cell number	The number of cells with lipid droplet smaller than 2 μm in lipid droplet diameter	The percentage of cells with lipid droplet smaller than 2 μm in lipid droplet diameter
WT	50	1	2
1	50	1	2
2	50	2	4
3	50	9	18
4	50	3	6
5	50	4	8
6	50	3	6
7	50	7	14
8	50	5	10
9	50	5	10
10	50	50	100
11	50	50	100
12	50	50	100
13	50	2	4
14	50	2	4
15	50	1	2
16	50	50	100
17	50	44	88
18	50	1	2
19	50	1	2
20	50	3	6
21	50	50	100
22	50	50	100

23	50	1	2
24	50	50	100
25	50	1	2

Supplementary Figures

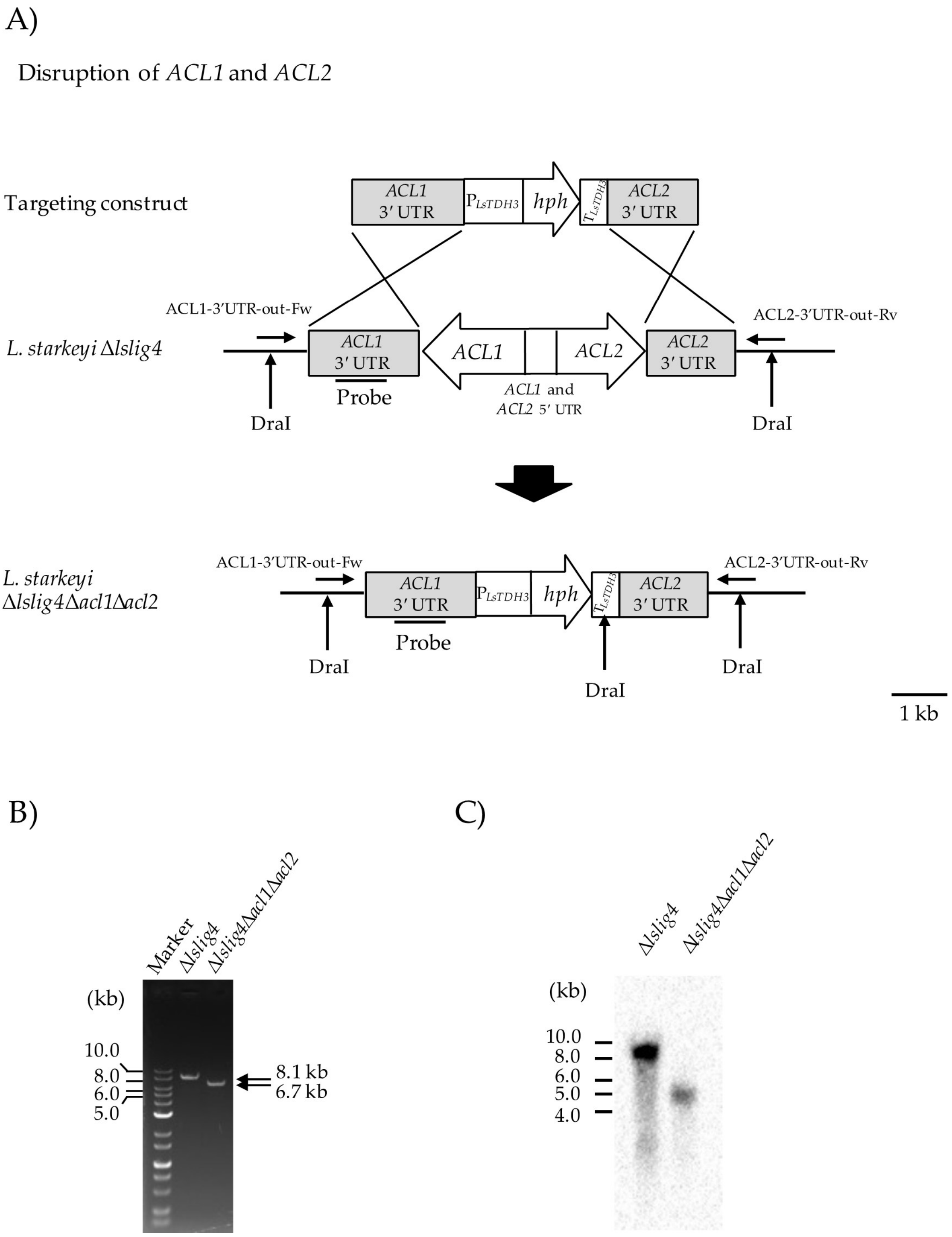

D)

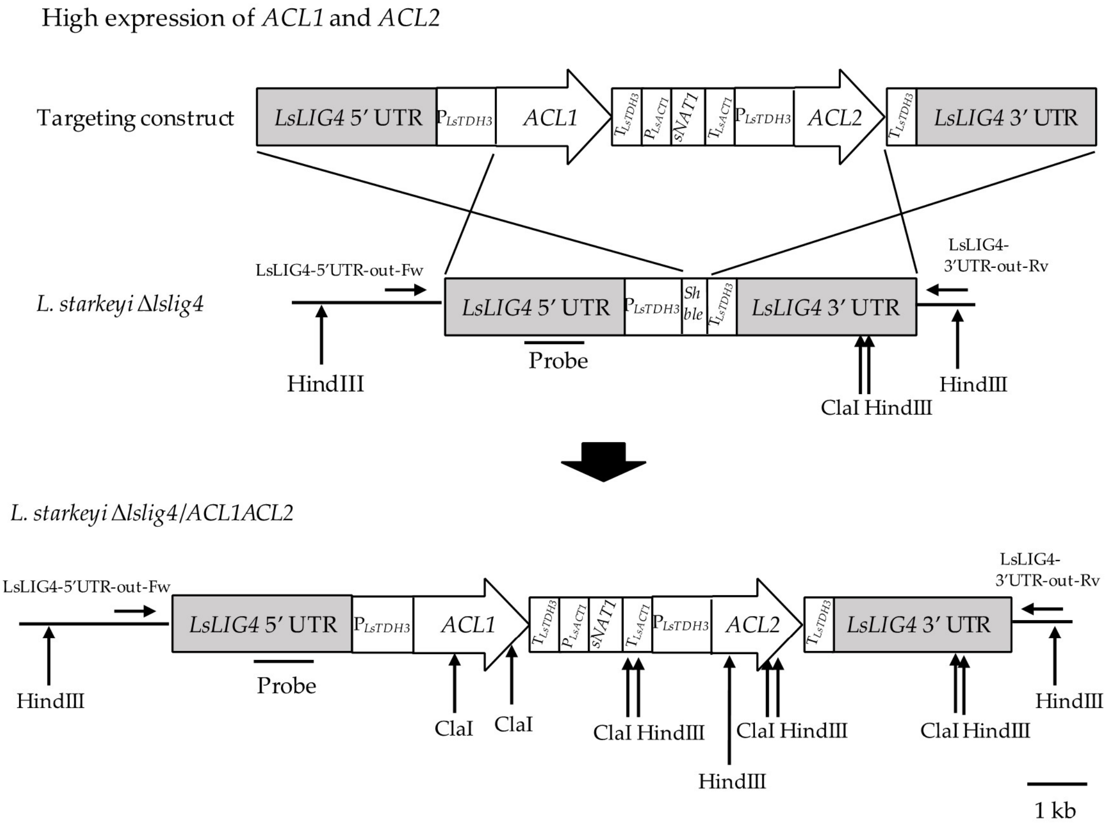

High expression of *ACL1* and *ACL2*
Targeting construct
LsLIG4 5′ UTR
P*LsTDH3*
ACL1
T*LsTDH3*
P*LsACT1*
sNAT1
T*LsACT1*
P*LsTDH3*
ACL2
T*LsTDH3*
LsLIG4 3′ UTR
L. starkeyi Δ*lslig4*
LsLIG4-5′UTR-out-Fw
LsLIG4-3′UTR-out-Rv
Sh ble
Probe
HindIII
ClaI HindIII
L. starkeyi Δ*lslig4*/*ACL1ACL2*
ClaI
1 kb

E)

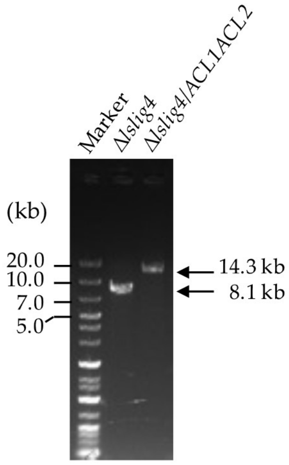

Marker
Δ*lslig4*
Δ*lslig4*/*ACL1ACL2*
(kb)
20.0
10.0
7.0
5.0
14.3 kb
8.1 kb

F)

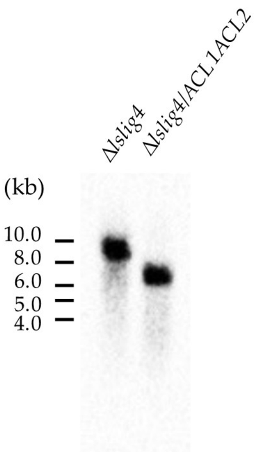

Δ*lslig4*
Δ*lslig4*/*ACL1ACL2*
(kb)
10.0
8.0
6.0
5.0
4.0

G)

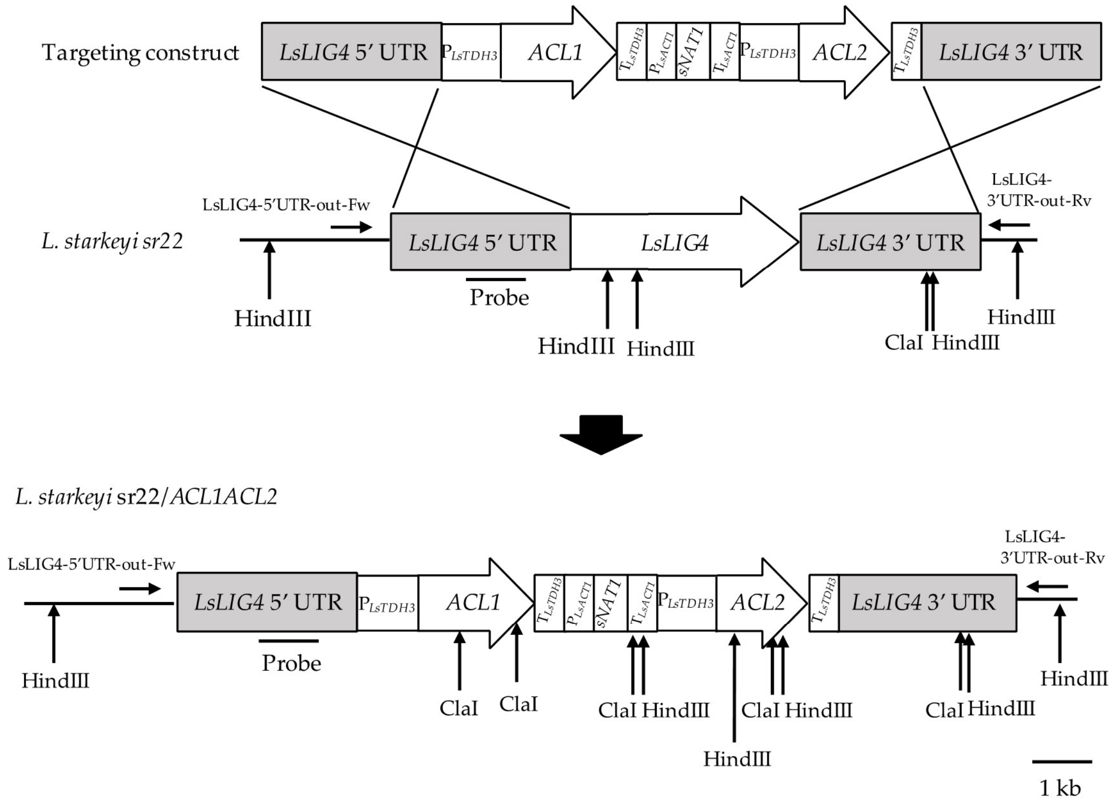

H)

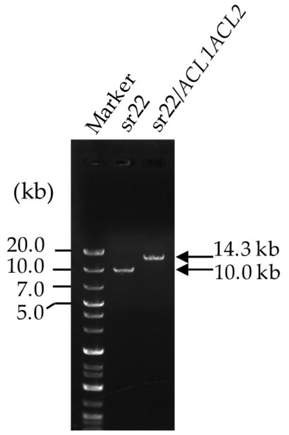

I)

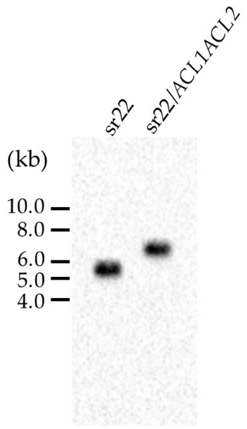

Figure S1. PCR and Southern blot confirmations of *ACL1* and *ACL2* deletion or high-expression. A) Strategy of the PCR and Southern blot analysis for the verification of the disruption of *ACL1* and *ACL2* in Δ*lslig4*. B) Confirmation of the replacement of the *ACL1* and *ACL2* ORF region by the *LsTDH3* promoter/*hph* ORF region/*LsTDH3* terminator DNA

fragment by PCR. The cultured cells on YPD agar plate containing 100 μg/mL hygromycin B was suspended with 100 μL of Lysing buffer containing 2% Triton X-100 and 1% SDS. The resultant cells were added 100 μL of phenol-chloroform after incubated 30 min at 70°C. After centrifugation at 15,000 × g for 5 min at room temperature, the supernatant was used as a template. PCR amplification was performed with primer set ACL1-3′UTR-out-Fw/ACL2-3′UTR-out-Rv and showed distinct bands representing different sizes 8.1 and 6.7 kb. The replacement event results in a 6.7-kb PCR product. C) genomic DNA of the indicated strains was digested with DraI, separated on 0.6% agarose gel, transferred to the Nylon membrane (BIODYNE B, Nihon Pall, Tokyo, Japan), and hybridized with the amplified specific probe (3′ UTR region of *ACL1*) using primer set ACL1ACL2-probe-Fw2/ACL1ACL2-probe-Rv2 in panel A). The *ACL1* and *ACL2*::*hph* fragment was detected by a 5.0 kb band, as opposed to a 8.8 kb band of the *ACL1* and *ACL2* genomic DNA fragment. D, G) Strategy of the PCR and Southern blot analysis for the verification of the high-expression of *ACL1* and *ACL2* in Δ*lslig4* or sr22. E, H) Confirmation of the replacement of the *ACL1*/*LsTDH3* terminator/*LsACT1* promoter/*sNAT1* ORF region/*LsACT1* terminator/*LsTDH3* promoter/*ACL2* DNA fragment by the *Sh ble* ORF region by PCR. The cultured cells on YPD agar plate containing 30 μg/mL nourseothricin was suspended with 100 μL of Lysing buffer containing 2% Triton X-100 and 1% SDS. The resultant cells were added 100 μL of phenol-chloroform after incubated 30 min at 70°C. After centrifugation at 15,000 × g for 5 min at room temperature, the supernatant was used as a template. PCR amplification was performed with primer set LsLIG4-5′UTR-out-Fw/LsLIG4-3′UTR-out-Rv and showed distinct bands representing different sizes 8.1, 14.3, 10.0 and 14.3 kb in Δ*lslig4*, Δ*lslig4*/*ACL1ACL2*, sr22 and sr22/*ACL1ACL2*, respectively. The replacement events resulted in a 14.3-kb PCR product. F, I) genomic DNA of the indicated strains was digested with ClaI and HindIII, separated on 0.6% agarose gel, transferred to Nylon membrane (BIODYNE B), and hybridized with the amplified specific probe (5′ UTR region of *LsLIG4*) using primer set LsLIG4-5′UTR-probe-Fw/LsLIG4-5′UTR-probe-Rv in a panel D and G). The *sh ble*::*ACL1*-s*NAT1*-*ACL2* fragment was detected by a 6.8 kb band in Δ*lslig4*/*ACL1ACL2*, as opposed to 9.0 kb band of the *LsLIG4* genomic DNA fragment. The *sh ble*::*ACL1*-s*NAT1*-*ACL2* fragment was detected by a 6.8 kb band in sr22/*ACL1ACL2*, as opposed to 5.6 kb band of the sr22 genomic DNA fragment.

A)

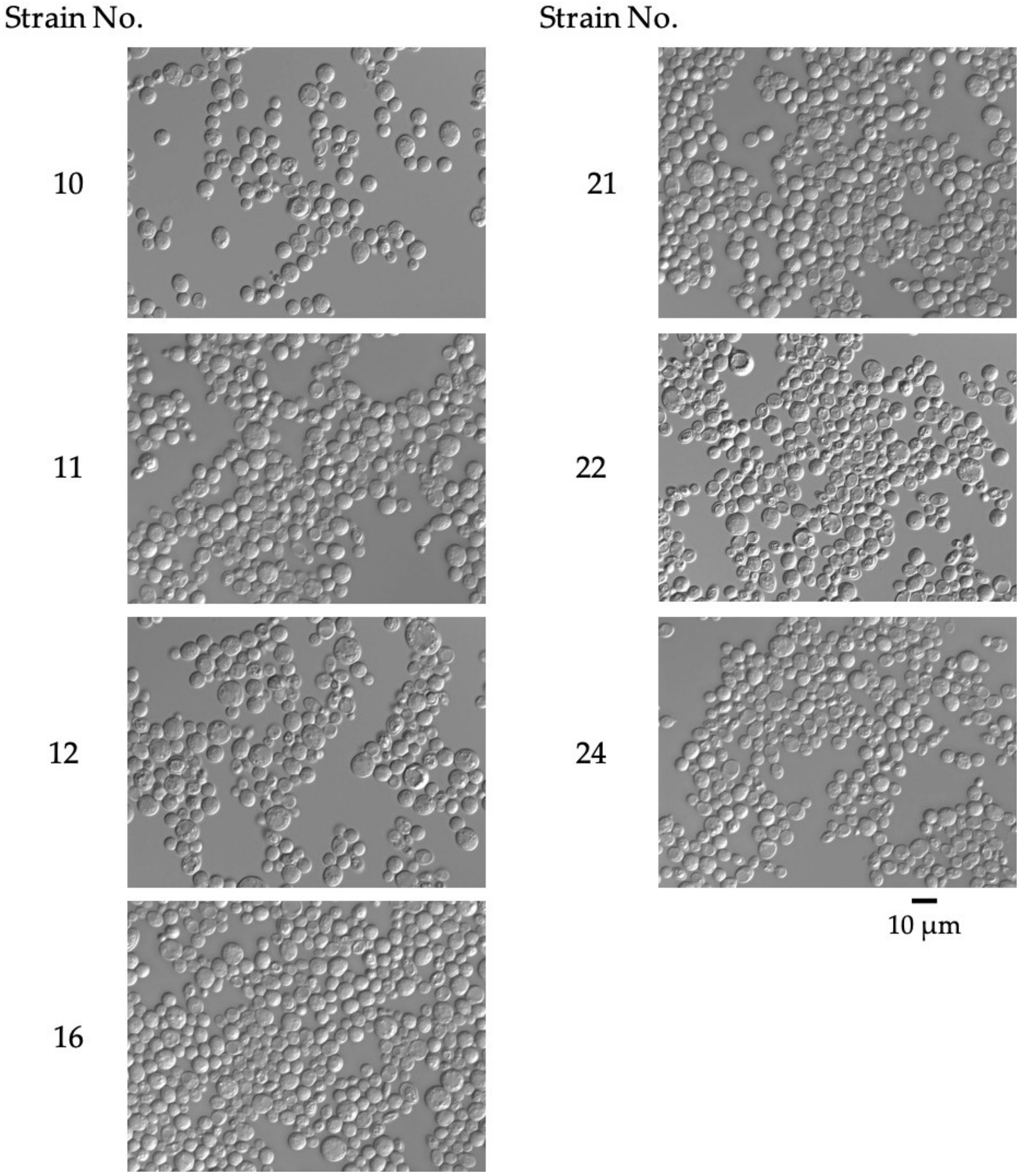

B)

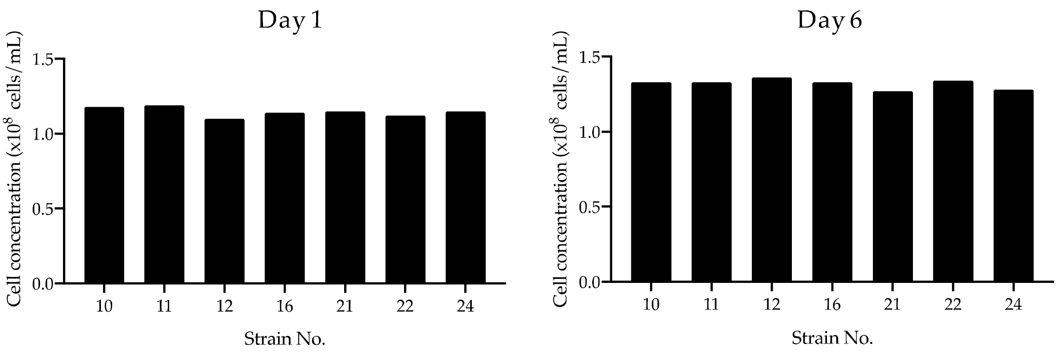

C)

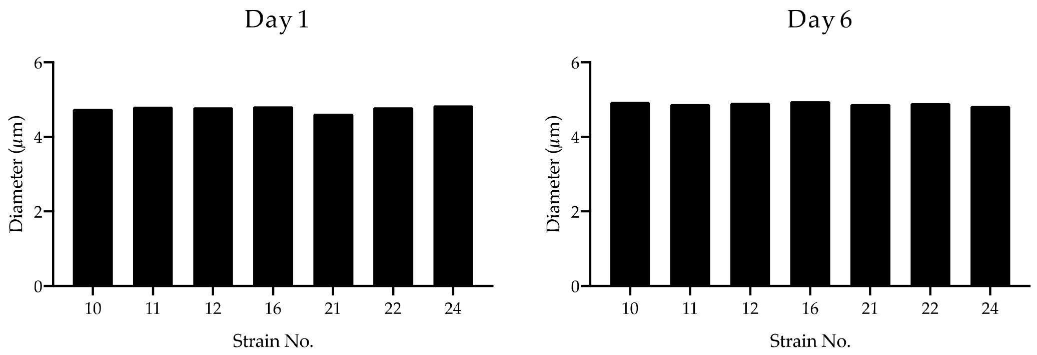

Figure S2. Phenotypes of mutant with greatly decreased lipid productivity strains (No. 10, 11, 12, 16, 21, 22, 24). Those mutants were cultured on S medium under large-scale cultivation condition at 30°C for six days. A) Differential interference contrast (DIC) images on day 6, B) cell concentration (cells/mL) on day 1 and 6, C) Diameter (μm) on day 1 and 6.

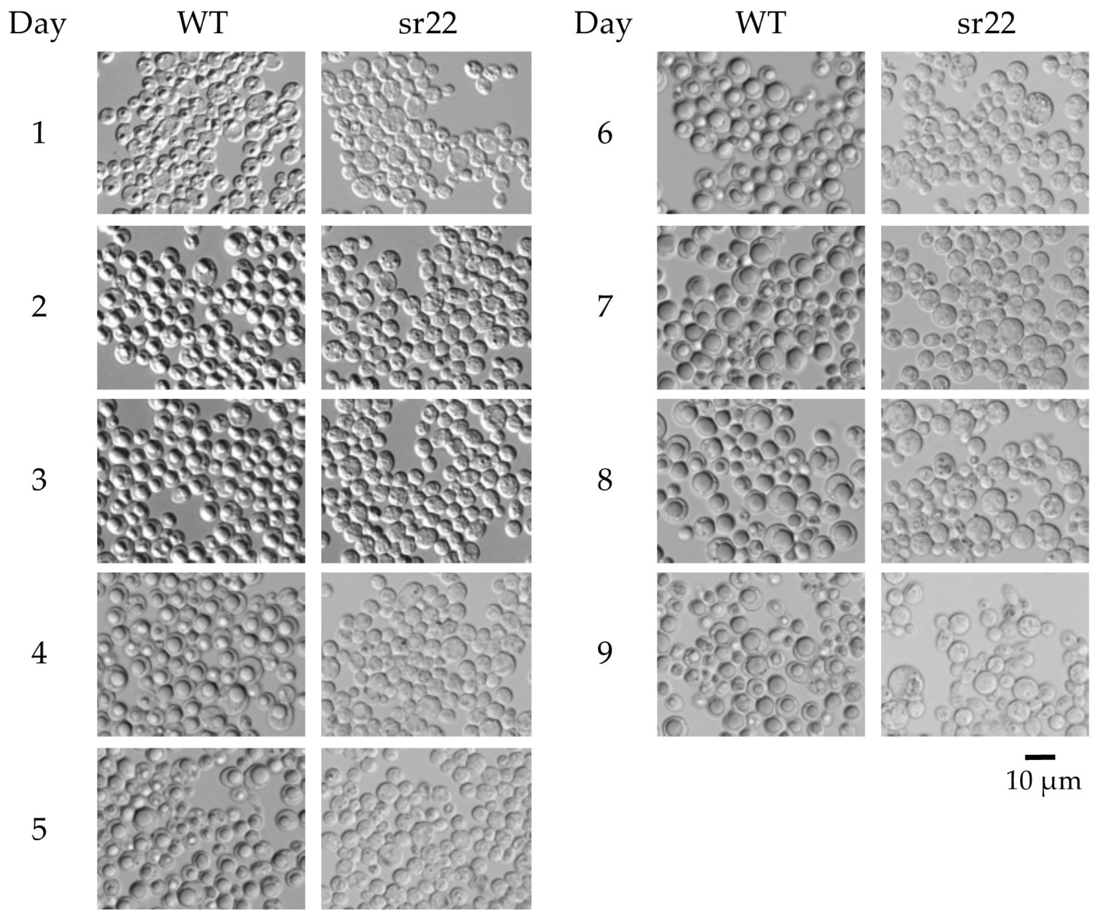

Figure S3. Substantial differences in DIC images of lipid droplets between wild-type (WT) strain and sr22 mutant. WT and sr22 cells were cultured on S medium under large-scale cultivation condition at 30°C for nine days.

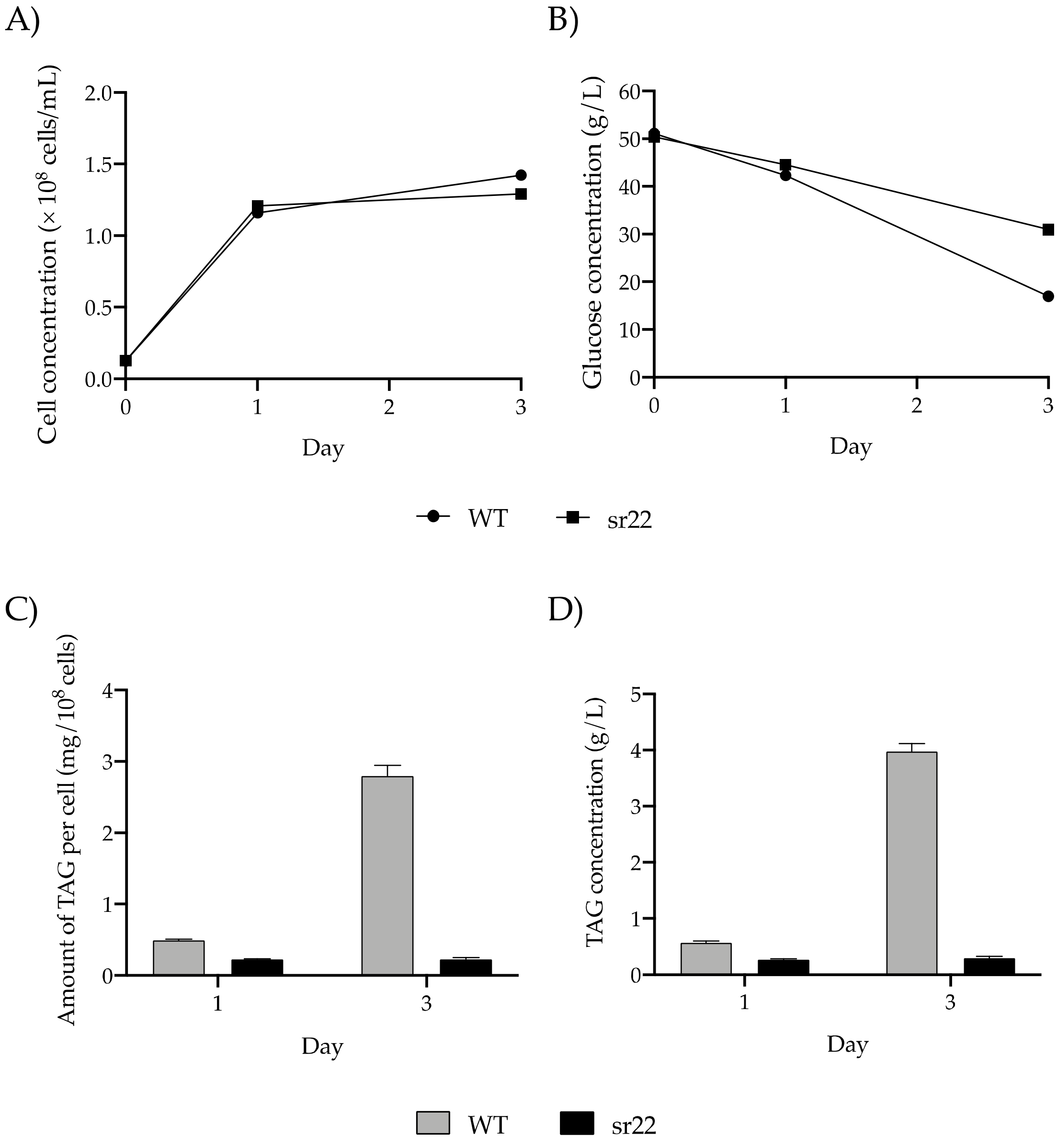

Figure S4. Time course analysis of cell concentration, glucose concentration, amount of TAG per cell, and TAG concentration in the wild-type (WT) strain and sr22 mutant. WT and sr22 were cultured on S medium under large-scale cultivation condition at 30°C for three days. A) cell concentration (cells/mL), B) glucose concentration (g/L), C) amount of TAG per cell (mg/10^8 cells), and TAG concentration (g/L). Data are indicated as the mean ± SEM of the three independent experiments.

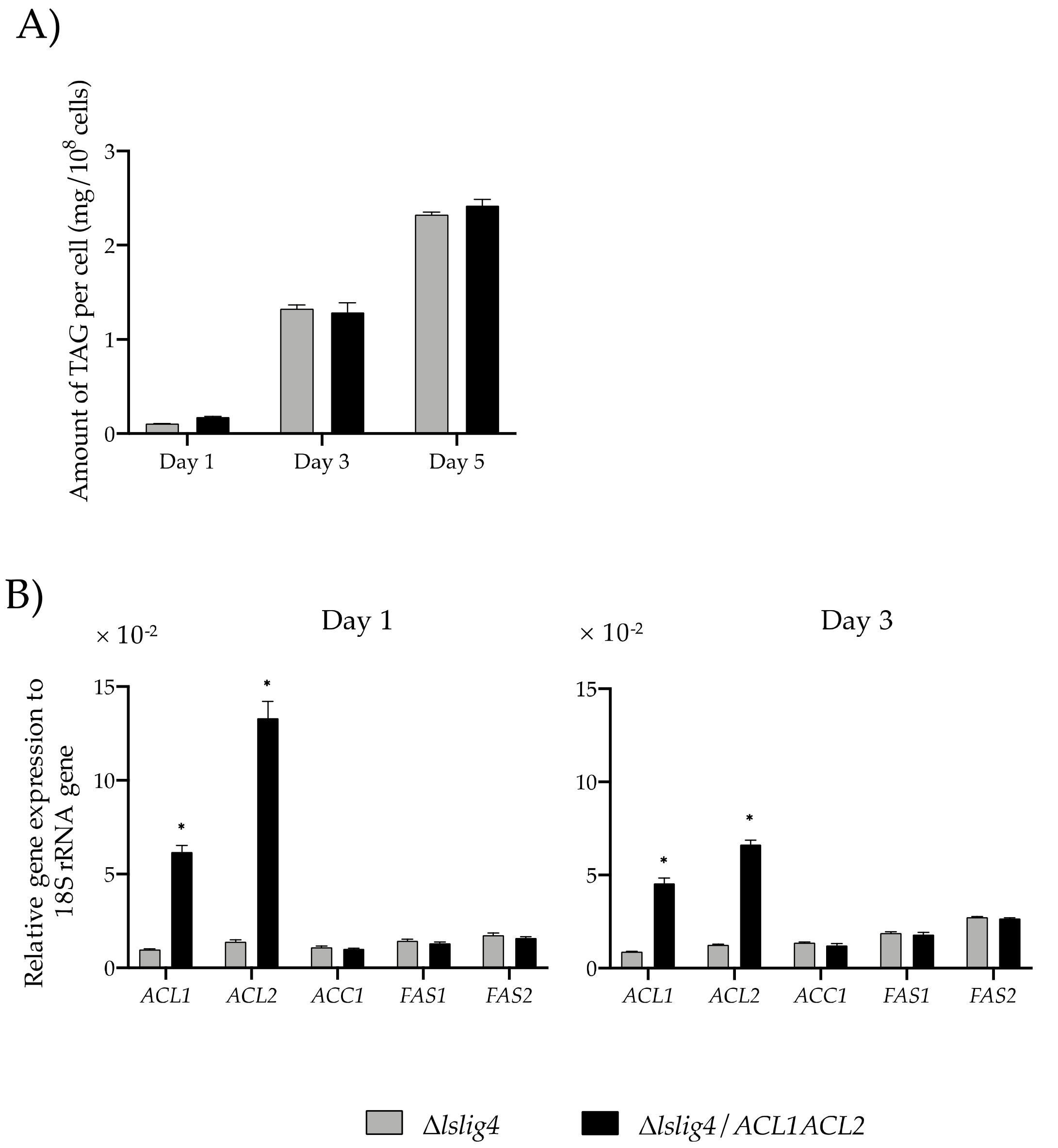

Figure S5. The effect of ATP-citrate lyase overexpression on TAG synthesis on an S medium containing glucose. Δ*lslig4* and Δ*lslig4*/*ACL1ACL2* were cultured on S medium under small-scale cultivation condition at 30°C for five days. Symbols: gray bars, Δ*lslig4*; black bars, Δ*lslig4*/*ACL1ACL2*. A) amount of TAG per cell (mg/10^8 cells), B) citrate-mediated acyl-CoA synthesis related genes. The transcription levels with relative quantitative method normalized by 18S rRNA. Data are indicated as the mean ± SEM of the three independent experiments. Asterisks indicate statistically significant differences between the Δ*lslig4* and Δ*lslig4*/*ACL1ACL2* (t test, *$P < 0.05$).

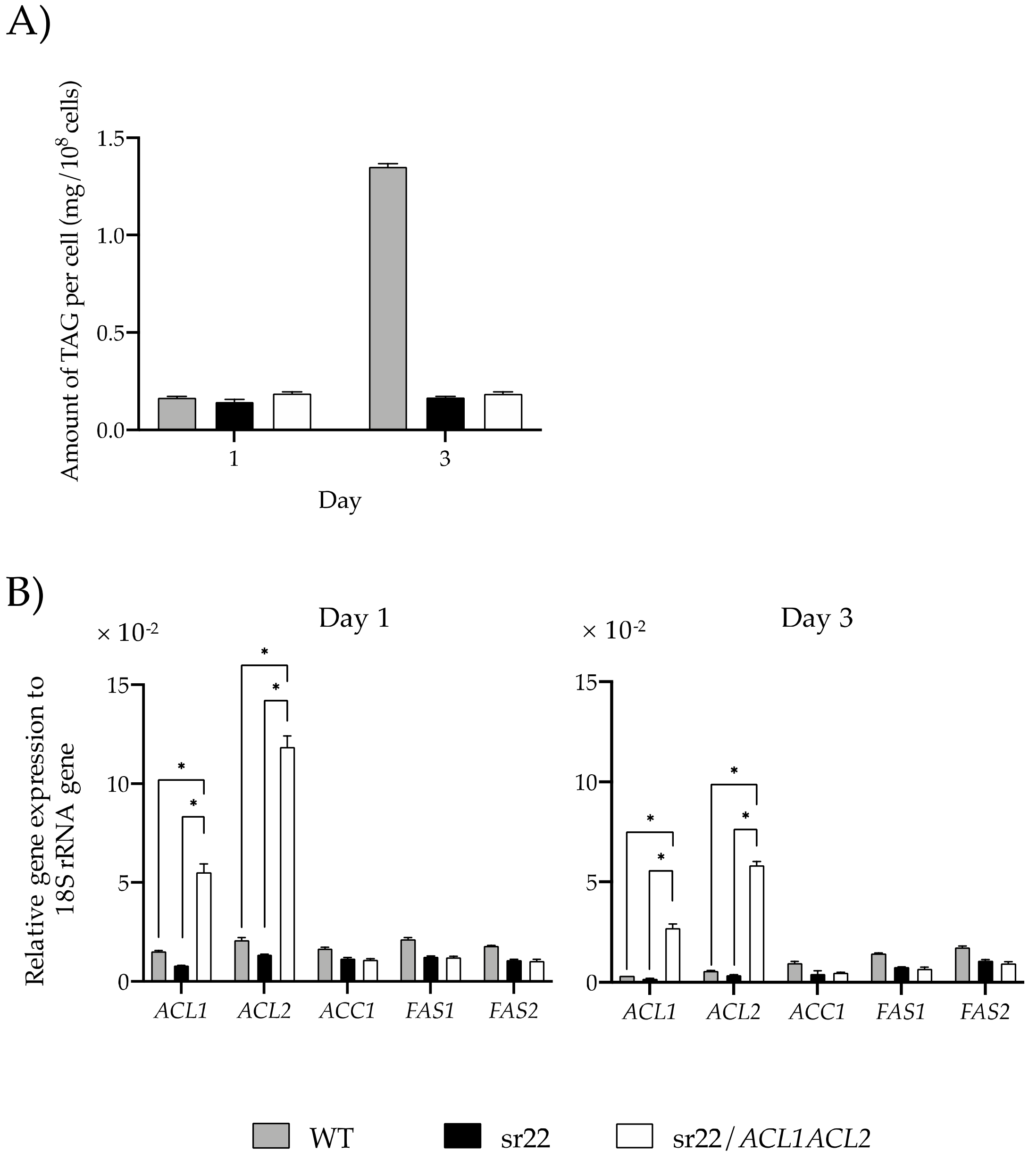

Figure S6. The effect of ATP-citrate lyase overexpression on TAG synthesis on an S medium containing glucose in sr22 mutant. Wild-type (WT), sr22 and sr22/*ACL1ACL2* were cultured on S medium under small-scale cultivation condition at 30°C for three days. Symbols: gray bars, WT; black bars, sr22; white bar, sr22/*ACL1ACL2*. A, B) the analysis of amount of TAG per cell (mg/10^8 cells) and relative expression levels of genes involved in citrate-mediated acyl-CoA synthesis was performed as described in Figure S5. Asterisks indicate statistically significant differences between the WT, sr22 and sr22/*ACL1ACL2* (t test, *$P < 0.05$).
